# The pCri System: A Vector Collection for Recombinant Protein Expression and Purification

**DOI:** 10.1371/journal.pone.0112643

**Published:** 2014-11-11

**Authors:** Theodoros Goulas, Anna Cuppari, Raquel Garcia-Castellanos, Scott Snipas, Rudi Glockshuber, Joan L. Arolas, F. Xavier Gomis-Rüth

**Affiliations:** 1 Proteolysis Lab, Molecular Biology Institute of Barcelona, CSIC, Barcelona Science Park, Helix Building, Barcelona, Spain; 2 Sanford-Burnham Medical Research Institute, La Jolla, California, United States of America; 3 Institute of Molecular Biology and Biophysics, Department of Biology, Zurich, Switzerland; Indian Institute of Science, India

## Abstract

A major bottleneck in structural, biochemical and biophysical studies of proteins is the need for large amounts of pure homogenous material, which is generally obtained by recombinant overexpression. Here we introduce a vector collection, the pCri System, for cytoplasmic and periplasmic/extracellular expression of heterologous proteins that allows the simultaneous assessment of prokaryotic and eukaryotic host cells (*Escherichia coli*, *Bacillus subtilis*, and *Pichia pastoris*). By using a single polymerase chain reaction product, genes of interest can be directionally cloned in all vectors within four different rare restriction sites at the 5′end and multiple cloning sites at the 3′end. In this way, a number of different fusion tags but also signal peptides can be incorporated at the N- and C-terminus of proteins, facilitating their expression, solubility and subsequent detection and purification. Fusion tags can be efficiently removed by treatment with site-specific peptidases, such as tobacco etch virus proteinase, thrombin, or sentrin specific peptidase 1, which leave only a few extra residues at the N-terminus of the protein. The combination of different expression systems in concert with the cloning approach in vectors that can fuse various tags makes the pCri System a valuable tool for high throughput studies.

## Introduction

Researchers performing biochemical, biophysical and biological studies on proteins commonly require large amounts of pure homogeneous material, which cannot usually be purified from natural sources. Alternatively, proteins are over-expressed heterologously in various systems incorporating host cells of bacterial, yeast, insect, or mammalian origin [1]–[3]. A critical step in protein production, after target selection, is to examine as many parameters as possible and to identify the most promising strategy for protein expression and purification with a minimum of resources and time.

Prior information on the protein of interest is crucial. An extensive search in databases such as NCBI (http://www.ncbi.nlm.nih.gov), UniProt (http://www.uniprot.org) and PDB (http://www.pdb.org) for known homologous proteins may identify possible problems and appropriate solutions for subsequent experiments. In addition, it is advisable to test protein orthologs of different origin, including distantly related or unrelated species (bacteria, archaea, and eukaryotes). At this point, analysis of the primary and secondary structure of both the encoding mRNA and the translated polypeptide may anticipate downstream problems.

There is a plethora of freely available software and databases for identifying protein families and sequence conservation patterns (PFAM) [4], putative signal peptides (SPs; SignalIP) [5], lipoboxes (DOLOP) [6], glycosylation, phosphorylation and other posttranslational modifications [7], transmembrane domains (TOPCONS, TMHMM, BOCTOPUS) [8]–[10], and unfolded/disordered regions (DisEMBL, PONDR, PSIPRED Protein Sequence Analysis Workbench) [11]–[13]. Protein location within the cell, i.e. cytoplasmic, periplasmic, or extracellular (PSORT, http://psort.hgc.jp), provides an indication of the requirements of the protein for proper folding, including disulfide bond formation and the need for special chaperons in each cellular compartment [14]–[16]. Further prediction of the secondary structure content (JPRED, LOMETS) [17], [18] can give clues about possible protein domains and motifs, a characterisation which may prove useful for chopping full-length multi-domain proteins into globular moieties. In general, successful recombinant protein expression depends on the removal of wild-type SP, lipoboxes, posttranslational signals, low-complexity regions, hydrophobic residues at the protein termini and membrane spanning regions, while conserving the boundaries of globular domains [19].

In parallel, cDNA characterisation is important in designing the cloning strategy and identifying potential problems at the transcriptional and translational levels. Although these processes are affected by a number of exo- and endo-nucleases, the stability of the resulting mRNA is critical in protein expression experiments [20]. mRNA can be protected by introducing sequences at the 5′ untranslated regions (UTRs) and stem loop structures at the 3′ UTRs [21]. The GC base content (>70%) may affect levels of expression and can be easily determined by sequence analysis software. Rare codons (GCUA 2.0) [22], especially consecutive ones, are frequently found in heterologous genes and may lead to translational errors due to ribosomal stalling [23], [24]. Such codon bias can be remedied by replacing selected codons or, if necessary, by overall gene optimisation using appropriate software (OPTIMIZER) [25]. Once the above requirements are fulfilled, the gene can be inserted into the vector by directional cloning using restriction enzymes that do not cut within the gene sequence (NEBcutter) [26]. Efficiency of translation termination can be increased by introducing strong stop codons (UAA, especially in context when followed by a U base, or consecutive ones) at the end of the translated gene [27]. Although present in many expression vectors, transcription terminators can be included downstream of the transcribed gene if instability is predicted [28]. Finally, sources of cDNA can be found in the Mammalian Gene Collection (http://mgc.nci.nih.gov/) and at the home page of Culture Collection of the World (http://www.ecotao.com/holism/agric/hpcc.html).

No expression system is generic for all target proteins, so both bacterial and eukaryotic systems need to be explored. *Escherichia coli* provides the cheapest expression host, and it is the most widely used but its machinery is not as sophisticated as that of eukaryotic hosts, and it cannot always express well folded proteins of variable origin [15]. Other alternatives often need to be tested, including bacterial systems such as *Bacillus subtilis* [29] and more advanced eukaryotic systems such as the yeasts *Pichia pastoris* [1] and *Saccharomyces cerevisiae* [30], the baculovirus expression system in insect cells [3], mammalian cells [31], or cell-free systems using prokaryotic extracts [32], which have highly variable cost-efficiency ratios.

With *E. coli* alone, many variables can be tested in order to improve expression levels and achieve proper protein folding [2], [33]. A number of specialised strains carrying mutations [34], [35] or plasmids that co-express proteins favouring expression at the transcriptional or translational level (e.g. pRARE or pLysE/pLysS) are available [24], [36]. Coupled expression of exogenous chaperones can assist in proper folding and prevent protein aggregation [37], [38]. Expression can also be influenced by other parameters, such as the culture method (e.g. batch fermentation, fed batch and dialysis fermentation) [39], cell growth media composition (lysogeny broth (LB), the enriched terrific broth (TB), two times yeast and tryptone broth (2×YT), and auto-induction media) [40], and culture conditions like temperature (18–37°C), shaking, aeration and other physical variables. All these factors can affect production levels, secretion, protein folding, solubility and host proteolytic activity [41], [42].

The many systems for introducing fusion tags currently available were originally developed to facilitate the detection and purification of recombinant proteins. Tags such as polyhistidine (His_6_-tag) and streptavidin-binding peptide (Strep-tag) allow purification by affinity chromatography and protein detection by Western blotting [43], [44], and others such as C-terminally fused green fluorescent protein (GFP) are an indispensable tool for membrane protein biochemists [45]. Finally, several studies have shown that the introduction of tags at the N- or C-terminus of proteins can improve expression levels by providing an optimized environment for translation initiation and mRNA protection, protein solubility [46]–[48], and carrier-driven crystallisation experiments [49].

Here we present a collection of vectors with which various expression systems and fusion tags can be evaluated simply and effectively. We examine the applicability of this system and provide several test cases, which support its robustness and versatility. This vector collection, which has been extensively tested and modified, is freely available to the scientific community under Addgene (https://www.addgene.org).

## Materials and Methods

### Genetic manipulations and vector preparation

Three series of vectors were generated on the basis of vectors available from the European Molecular Biology Laboratory (pETMBP-1a, pETTRX-1a, and pETGST), Novagen (pET-26b, and pET-28a), MoBiTec (pHT-01, and pHT-43), Invitrogen (pPICZA and pPICZαA), and from the Glockshuber laboratory (pRBI-DsbC) [50]. The inserted sequences for pCri-11, 13, and 14 were amplified from pET-15b-SUMO1 [51], pMIS3.0E [52], and pKLSLt [53], respectively. All vectors were prepared for directional cloning in *Nco*I or *Nde*I restriction sites at the 5′end and in *Xho*I at the 3′end. The gene coding for GFP (UniProt code: B6UPG7; 729 bp), including a multiple cloning site (MCS; from pETMBP-1a; 52 bp), was introduced into all vectors. The insert was cloned between the *Nco*I or *Nde*I and *Xho*I restriction sites and was modified to contain an *Msc*I or *Nhe*I restriction site immediately after the *Nco*I and *Nde*I sites, respectively. Standard cloning techniques were used throughout [54]. Polymerase chain reaction (PCR) primers and DNA modifying enzymes were purchased from Sigma-Aldrich and Thermo-Scientific, respectively. PCR was performed using Phusion high-fidelity DNA polymerase (Thermo-Scientific) according to the manufacturer's instructions and following a standard optimisation step of a thermal gradient in each reaction. For vector preparation, a number of insertions and mutations introduced or eliminated nucleotide sequences. We followed a PCR-based strategy described elsewhere [55], including a *Dpn*I digestion step to remove parental DNA. Digestion with restriction enzymes was carried out according to standard protocols. When necessary, a second round of digestion was performed before the final DNA purification step. DNA was purified from PCR reactions, enzymatic reactions, agarose gel band extractions, and vector extractions using OMEGA-Biotek purification kits. Chemically competent *E. coli* DH5α, BL21 (DE3), and Origami 2 (DE3) cells (Novagen) were prepared and transformed following Hanahan method [56]. Competent cells of *P. pastoris* KM71H (Invitrogen) and *B. subtilis* WB800N (MoBiTec) were prepared according to the manufacturer's instructions.

### Protein expression and purification

For expression trials, *mecR1* (UniProt code: P0A0B0; an integral-membrane metallopeptidase) was cloned into vector pCri-8a and 13a; the gene coding for fragilysin (UniProt code: O86049; Ala212-Asp397; a soluble metalloendopeptidase) into pCri-1a, 4a, 6a and 8a; *gfp* into pCri-1a, 4a, 6a, 8a, 11a, and 14a; the gene coding for carboxypeptidase A2 (CPA2; UniProt code: P48052; Leu19-Tyr419; a soluble metalloexopeptidase) into pCri-8a, 9a, 16a, and 18a; and the gene coding for peptide-N-glycosidase F (PNGase F; UniProt code: P21163; Ala41-Asp354; a soluble glycosidase) into pCri-4a and 8a. The constructs were transformed in *E. coli* BL21 (DE3), Origami 2 (DE3), or *B. subtilis* cells and plated on LB plates supplemented with antibiotics (30 µg/mL kanamycin or 5 µg/mL chloramphenicol). A single colony was inoculated in 5 mL LB broth and incubated overnight at 30°C with stirring at 250 rpm. 1 mL of the pre-inoculum was used to inoculate 100 mL of LB broth and cells were left to grow at 37°C until OD_600 nm_≈0.7–0.8. Subsequently, cells were incubated with 0.4–1 mM isopropyl-β-D-1-thiogalactopyranoside (IPTG) to induce protein expression and kept for 5 h at 37°C or overnight at 20°C.

For expression trials in *P. pastoris* cells, vectors were linearized with *Pme*I restriction enzyme and transformed using the *Pichia* EasyComp transformation kit (Invitrogen). Cells were inoculated in low salt yeast peptone dextrose (YPD) plates supplemented with 100 µg/mL zeocin and incubated for 3–4 days at 28°C. Colonies were selected and grown in 100 mL buffered complex glycerol medium (BMGY) at 28°C until an OD_600 nm_≈2. Cells were then harvested, resuspended in buffered complex methanol medium (BMMY), and protein expression was induced with 0.5% methanol.

Cells were separated from the growth media by centrifugation at 8,000×*g* for 30 min at 4°C. Secreted proteins were collected from the growth media and dialysed in buffer A (50 mM Tris-HCl, 250 mM NaCl, pH 7.5), and cytoplasmic proteins were extracted from the cells in the same buffer. For lysis, cells were sonicated with 3 pulses of 5 min each at 40% amplitude (Branson digital sonifier). Samples were collected before and after centrifugation (30,000×*g* for 30 min at 4°C) representing total and soluble protein fractions, respectively.

Selected samples were further purified by affinity chromatography using either nickel-nitrilotriacetic acid- (Ni-NTA), maltose binding protein- (MBP) or glutathione S-transferase- (GST) HiTrap columns, or a Sepharose 4B matrix column (GE Healthcare Life Sciences). 10 mL of crude protein extract was applied to the columns, followed by three washes with buffer A. Proteins were eluted with buffer A supplemented with either 300 mM imidazole (Ni-NTA-affinity), 10 mM maltose (MBP-affinity), 10 mM reduced glutathione (GST-affinity) or 20 mM lactose (Sepharose-affinity). Finally, samples were buffer-exchanged to buffer B (20 mM Tris-HCl, 150 mM NaCl, pH 7.4) using a PD-10 desalting column (GE Healthcare Life Sciences). Samples were kept at 4°C at all times.

For expression and purification of MecR1, the cultures were scaled up to 6L, the collected cells were broken with a cell disrupter (Constant Cell Disruption Systems) at 2.4kBar and non-disrupted cells and cell debris were removed by centrifugation at 20,000×*g* for 45 min in a Sorvall centrifuge. Membranes were collected by ultracentrifugation at 150,000×*g* for 2 h at 4°C in a Beckman Optima L-90K using a 50.2 Ti rotor (Beckman) and 26.3-ml polycarbonate bottles with cap assembly (Beckman). Collected membranes were homogenized using a glass Potter and solubilized under gentle stirring by overnight incubation at 4°C in buffer C (50 mM Tris-HCl, 300 mM NaCl, 10 mM imidazole, 1 mM 1,4-dithio-D-threitol, pH 8.0) containing 100 mM lauryl-dimethylamine N-oxide (LDAO; Sigma) and EDTA-free proteinase inhibitor cocktail tablets (Roche). Non-solubilized proteins were removed by ultracentrifugation as described above. The sample was incubated overnight at 4°C with Ni-NTA resin (Invitrogen). The bound protein was batch purified in an open column (Bio-Rad), washed extensively, and the tagged protein eluted with buffer C plus 300 mM imidazole. The sample was desalted using a PD-10 column in buffer C containing 5 mM LDAO.

### Fusion-tag removal by proteinase cleavage

Tobacco etch virus (TEV) proteinase and sentrin specific proteinase 1 (SENP1) were over-expressed in *E. coli* BL21 (DE3) pLysE cells using pET28-based vectors, which attach an N-terminal His_6_-tag. Cultures (typically 4L) were grown in LB broth at 37°C until an OD_600 nm_≈0.7–0.8, induced with 0.5 mM IPTG, and incubated either overnight at 20°C or for 5 h at 30°C for TEV proteinase or SENP1 expression, respectively. Subsequently, cells were collected by centrifugation at 5,000×*g* for 30 min at 4°C and partially purified by Ni-NTA affinity chromatography as previously described [57], [58]. Proteinases were stored at −80°C in buffer D (20 mM Tris-HCl, 50 mM NaCl, pH 7.5, 30% glycerol). Proteinase cleavage trials of tagged-proteins were performed overnight at 4°C in buffer B using various protein∶proteinase ratios. For trials with thrombin (GE Healthcare Life Sciences), 2 units of proteinase were used to process 25 µg of protein in 100 µL of buffer C at room temperature and aliquots were taken at various time points.

### Enzymatic assays

For hydrolytic activity measurements, PNGase F and fragilysin were partially purified by Ni-NTA-affinity chromatography as described above. Glycosidase activity of PNGase F was tested against the glycoprotein ribonuclease B (RNase B; New England Biolabs) at a w/w ratio of 1∶5 PNGase F/RNase B and a final protein concentration of 0.5 mg/mL. Reactions were incubated overnight at 4°C and analysed by SDS-PAGE. Peptidase activity of fragilysin was tested against BODIPY FL-casein (Invitrogen) as previously described [59]. Crude protein extracts of CPA2 were used for assays after an initial activation with partial tryptic digestion in a w/w ratio of 1/100 of CPA2/trypsin at room temperature for 1 h. The activated protein was incubated with furyl-acryloyl-L-phenylalanine-L-phenylalanine (0.05 mM; Sigma) in buffer B and the activity was monitored by measurement of the absorbance change at 330 nm.

### Western-blot analysis

Protein samples were analyzed by Tricine-SDS-PAGE, transferred to Hybond ECL membranes (GE Healthcare Life Sciences), and finally blocked overnight at room temperature with 20 mL of blocking solution (137 mM NaCl, 2.7 mM KCl, 4.3 mM Na_2_HPO_4_, 1.47 mM NaH_2_PO_4_, 0.05% Tween 20) containing 1.5% bovine serum albumin. MecR1 was detected by immunoblot analysis using custom polyclonal antibodies (Eurogentec) at dilution 1∶1,000 and a secondary antibody (goat anti-rabbit IgG (HL) peroxidase-conjugated antibody; Pierce) at dilution 1∶5,000 (both in blocking solution). The immune complexes were detected using an enhanced chemiluminescence system (Super Signal West Pico Chemiluminescent; Pierce) according to the manufacturer's instructions. Membranes were exposed to hyperfilm ECL films (GE Healthcare Life Sciences).

### Miscellaneous

Denatured protein samples were analyzed by 10%–15% Tricine-SDS-PAGE [60] and stained with Coomassie-brilliant blue. Protein concentrations were routinely determined by absorbance at 280 nm, and, wherever necessary, corrected by the BCA protein assay method (Thermo Scientific) using bovine serum albumin as a standard. Protein identification by peptide mass fingerprinting was performed at the Protein Chemistry Facility of Centro de Investigaciones Biológicas (Madrid, Spain). Figures of vector maps were prepared with GENEIOUS (Biomatters).

## Results and Discussion

### Description of the pCri System

We generated a collection of vectors for recombinant protein overexpression in two bacterial (*E. coli* and *B. subtilis*) and one eukaryotic (*P. pastoris*) host strains. Vectors, available from commercial sources or laboratories, were initially modified by inserting new nucleotide sequences or point mutations, and finally evaluated for functionality. Most of the *E. coli* vectors are pET based [61] with the exception of pCri-12, which is based on pTrc99a [50]. The bacillus and yeast vectors are based on pHT [62] and pPICZ series [63], respectively, and can be stably propagated in *E. coli* cells when antibiotic resistance is conferred (Tables 1–3). In all vectors, protein expression is achieved by IPTG induction, except for the yeast vectors, for which methanol is required.

**Table 1:** pCri-a vectors.

Name	Vector size (∼kbp) a	Fusion tag b	Extra residues after fusion removal	Fusion molecular mass (∼kDa) c	Resistance marker d	Based on vector	Cells
pCri-1a	7.2	**His_6_-MBP-TEV-**insert**-His_6_-tag**	Gly-Ala	43.1	Kan^r^	pETMBP-1a	*E. coli*
pCri-4a	6.4	**His_6_-TRX-TEV-**insert**-His_6_-tag**	Gly-Ala	14.3	Kan^r^	pETTrx-1a	*E. coli*
pCri-6a	6.7	**His_6_-GST-TEV-**insert**-His_6_-tag**	Gly-Ala	28.9	Kan^r^	pETGST-1a	*E. coli*
pCri-7a	6.0	insert**-His_6_-tag**	-	-	Kan^r^	pET-28a	*E. coli*
pCri-8a	6.1	**His_6_-TEV-**insert**-His_6_-tag**	Gly-Ala	2.4	Kan^r^	pET-28a	*E. coli*
pCri-9a	6.1	**SP** (pelB)-insert**-His_6_-tag**	-	-	Kan^r^	pET-26b	*E. coli*
pCri-11a	6.3	**His_6_-SUMO-**insert**-His_6_-tag**	Ala	12.6	Kan^r^	pET-26b	*E. coli*
pCri-12a	5.2	**SP** (ompA)-insert**-His_6_-tag** and DsbC coexpression	-	-	Amp^r^	pRBI-DsbC	*E. coli*
pCri-13a	6.4	**His_8_-MISTIC-THR** e **-**insert**-His_6_-tag**	Gly-Ser-Gly_3_-Ala	16.2	Kan^r^	pET-28a	*E. coli*
pCri-14a	6.5	**His_6_-LSL-TEV-**insert**-His_6_-tag**	Gly-Ala	19.6	Kan^r^	pET-28a	*E. coli*
pCri-15a	4.0	**His_6_-TEV-**insert	Gly-Ala	2.4	Zeo^r^	pPICZA	*P. pastoris*
pCri-16a	4.4	**SP** (α-factor)-insert**-His_6_-tag**	-	-	Zeo^r^	pPICZαA	*P. pastoris*
pCri-17a^f^	8.6	**His_6_-TEV-**insert**-His_6_-tag**	Gly-Pro	2.4	Amp^r^, Cm^r^	pHT-01	*B. subtilis*
pCri-18a^f^	8.7	**SP** (samyQ)-insert**-His_6_-tag**	-	-	Amp^r^, Cm^r^	pHT-43	*B. subtilis*

a Calculated vector sizes based on the electrophoretic mobility with a Lambda DNA marker as standard.

b For introducing a His_6_-tag at the C-terminus of the target protein use a reverse primer without a stop codon.

c Molecular mass of C-terminal His_6_-tag ∼1 kDa.

d Kan^r^, 30–50 µg/mL; Amp^r^, 50–100 µg/mL; Cm^r^, 5 µg/mL; Zeo^r^, 25–100 µg/mL.

e Thrombin cleavage site (THR).

f Ampicillin resistance in *E. coli* and chloramphenicol resistance in *B. subtilis*.

**Table 2:** pCri-b vectors.

Name	Vector size (∼kbp) a	Fusion tag b	Extra residues after fusion removal	Fusion molecular mass (∼kDa) c	Resistance marker d	Based on vector	Cells
pCri-1b	7.2	**His_6_-MBP-TEV-**insert**-His_6_-tag**	Gly-His	43	Kan^r^	pCri-1a	*E. coli*
pCri-4b	6.4	**His_6_-TRX-TEV-** insert**-His_6_-tag**	Gly-Ala-His	14.3	Kan^r^	pCri-4a	*E. coli*
pCri-6b	6.7	**His_6_-GST-TEV-**insert**-His_6_-tag**	Gly-His	28.9	Kan^r^	pCri-6a	*E. coli*
pCri-7b	6.0	insert**-His_6_-tag**	-	-	Kan^r^	pCri-7a	*E. coli*
pCri-8b	6.1	**His_6_-tag-TEV**-insert**-His_6_-tag**	Gly-His	2.4	Kan^r^	pCri-8a	*E. coli*
pCri-9b	6.1	**SP** (pelB)-insert**-His_6_-tag**	-	-	Kan^r^	pCri-9a	*E. coli*
pCri-11b	6.3	**His_6_-SUMO-**insert**-His_6_-tag**	His	12.6	Kan^r^	pCri-11a	*E. coli*
pCri-12b	5.2	**SP** (ompA)-insert**-His_6_-tag** and DsbC coexpression	-	-	Amp^r^	pCri-12a	*E. coli*
pCri-14b	6.5	**His_6_-LSL-TEV-**insert**-His_6_-tag**	Gly-His	19.6	Kan^r^	pCri-14a	*E. coli*
pCri-15b	4.0	**His_6_-TEV-**insert**-His_6_-tag**	Gly-His	2.4	Zeo^r^	pCri-15a	*P. pastoris*

a Calculated vector sizes based on the electrophoretic mobility with a Lambda DNA marker as standard.

b For introducing a His_6_-tag at the C-terminus of the target protein use a reverse primer without a stop codon.

c Molecular mass of C-terminal His_6_-tag ∼1 kDa.

d Kan^r^, 30–50 µg/mL; Amp^r^, 50–100 µg/mL; Cm^r^, 5 µg/mL; Zeo^r^, 25–100 µg/mL.

**Table 3:** pCri-a-Strep vectors.

Name	Vector size (∼kbp) a	Fusion tag b	Extra residues after fusion removal	Fusion molecular weight (∼kDa) c	Resistance marker d	Based on vector	Cells
pCri-1a-Strep	7.2	**His_6_-MBP-TEV-**insert**-Strep-tag**	Gly-Ala	43.1	Kan^r^	pCri-1a	*E. coli*
pCri-4a-Strep	6.4	**His_6_-TRX-TEV-**insert**-Strep-tag**	Gly-Ala	14.3	Kan^r^	pCri-4a	*E. coli*
pCri-7a-Strep	6.0	insert**-Strep-tag**	-	-	Kan^r^	pCri-7a	*E. coli*
pCri-8a-Strep	6.1	**His_6_-TEV-**insert**-Strep-tag**	Gly-Ala	2.4	Kan^r^	pCri-8a	*E. coli*
pCri-9a-Strep	6.1	**SP** (pelB)-insert**-Strep-tag**	-	-	Kan^r^	pCri-9a	*E. coli*

a Calculated vector sizes based on the electrophoretic mobility with a Lambda DNA marker as standard.

b For introducing a Strep-tag at the C-terminus of the target protein use a reverse primer without a stop codon.

c Molecular mass of C-terminal Strep-tag ∼1.3 kDa.

d Kan^r^, 30–50 µg/mL.

**Figure 1:**
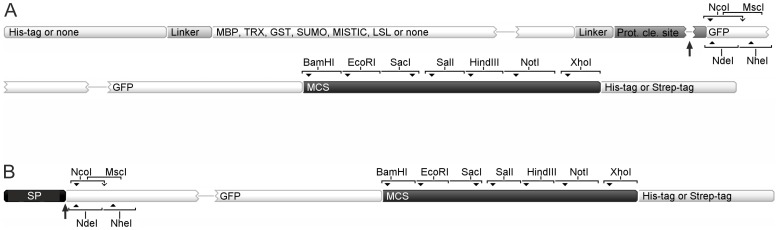
Vector overview of the pCri System. (**A**) Vectors for cytoplasmic protein expression. (**B**) Vectors for periplasmic and extracellular protein expression. An N-terminal His_6_-tag can be fused in all vectors for intracellular expression except of pCri-7. Other tags can also be fused including MBP, TRX, GST, SUMO, MISTIC, and LSL (Table 1–3). In all vectors, a C-terminal His_6_-tag or Strep-tag is attached if a stop codon is omitted within the target gene. Black arrows indicate the proteinase (i.e. TEV, SENP1 or thrombin) and signal peptide (SP) cleavage sites. Restriction sites allowing directional cloning are also shown. For more details regarding each vector, refer to Fig. S1.

The collection consists of 29 vectors grouped into three main categories (Tables 1–3). Based on the available 5′end restriction sites for target gene cloning, the vectors are sorted into pCri-a and pCri-b series using either *Nco*I and *Msc*I or *Nde*I and *Nhe*I sites, respectively (Fig. 1 and Fig. S1). The pCri-a series is further separated into pCri-a and pCri-a-Strep based on the fusion tag that can be attached at the C-terminus of the target protein. Within each category, the vectors allow obtaining constructs with different fusion tags or expression in a particular host organism. Usage of the aforementioned 5′end restriction sites incorporates a methionine start codon, thus obviating the need to introduce it into the target gene during PCR amplification. An MCS universal for all vectors has been placed at the 3′end, which encodes seven rare restriction sites not found in most of the vectors (see vector maps for more details; Fig. S1). For convenience and tracking during vector preparation, a GFP insert is cloned within all vectors. The inserted genes can be sequenced from either terminus with specific primers as detailed in Table 4.

**Table 4:** Sequencing primers for the pCri System.

Primer Name	Vector (pCri)	Sequence site	Primer sequence
T7 promoter	1-14a,b except 12a–b*	5′end	Universal
T7 terminator	1-14a,b except 12a–b*	3′end	Universal
seq-pCri-1	1a and b internal*	5′end	GAAATCATGCCGAACATCCC
seq-pCri-4	4a and b internal*	5′end	GCGGCAACCAAAGTGGGTGCAC
seq-pCri-6	6a and b internal*	5′end	GACCATCCTCCAACTAGTG
seq-pCri-11	11a and b internal*	5′end	CAAAAGAACTGGGAATG
5′seq-pCri-12	12a** and b**	5′end	GATAACGAGGGCAAAAAATG
3′seq-pCri-12	12a* and b*	3′end	CAAAGTAAACAACATAAAAC
seq-pCri-13	13a internal*	5′end	CAGATTTTATCCATCTC
seq-pCri-14	14a and b internal*	5′end	CTTCTGGAATCACCCTC
5′ AOX1	15a,b* and 16a**	5′end	Universal
3′ AOX1	15a,b* and 16a*	3′end	Universal
5′seq-pCri-17	17a* and 18a**	5′end	CTTATCACTTGAAATTG
3′seq-pCri-17	17a* and 18a*	3′end	GATTTTATTAGTACAGGGAC

*Hybridises before *Nco*I, *Nde*I or *Xho*I restriction sites.

**Hybridises before the SP.

Preparation is greatly simplified, as only two restriction sites are used for directional cloning of a target gene into a large series of vectors. Although newer cloning techniques are now available (e.g. ligation independent cloning system [64]), this method was satisfactory. Cloning of target genes of variable size spanning from 150 to 7,000 base pairs was routinely performed with a success rate of more than seven out of ten positive clones when genes were cloned between an *Nco*I or *Nde*I and a *Xho*I site. To achieve reproducible results, it was essential to repeat double digestions of the vectors with all the restriction enzyme combinations.

### Applications and main considerations of the pCri System

The choice and use of a suitable vector should be based on the properties of the target protein and the needs of the experiment in question. Here, in an effort to evaluate the functionality of the collection and to provide a rationale for the use of the vectors, we cloned and expressed several proteins of different origin and function:

#### Fusion tags assisting in protein purification

**Figure 2:**
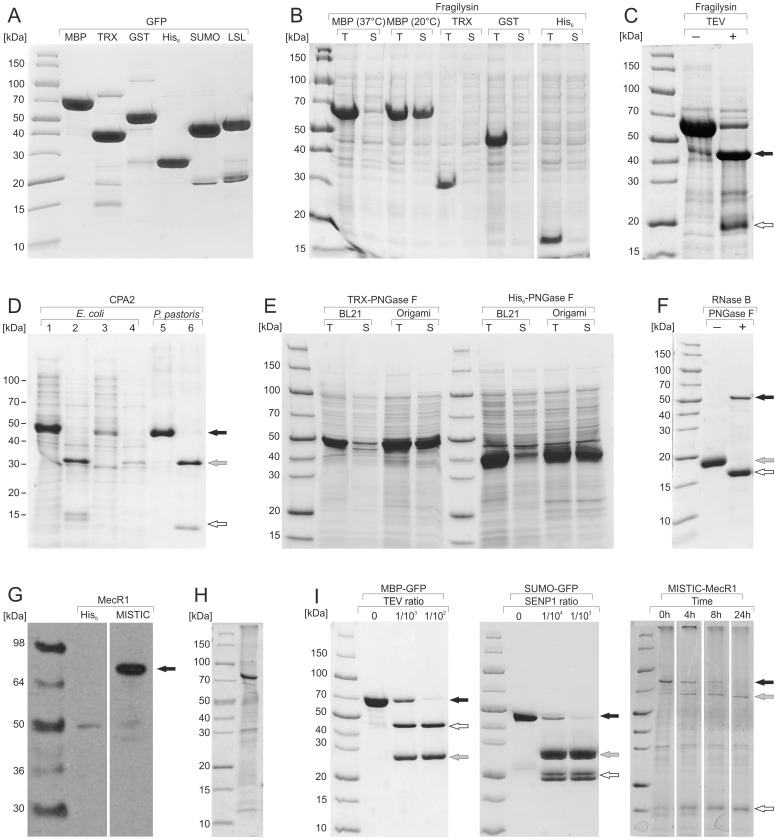
Protein expression and purification trials using the pCri System. (**A**) The GFP gene was cloned into pCri-1a, 4a, 6a, 8a, 11a, and 14a, the proteins expressed in *E. coli* BL21 cells, and subsequently purified by Ni-NTA-affinity chromatography except for MBP, GST, and LSL fusion products, which were purified by their respective specific affinity resins. (**B**) The gene coding for fragilysin was cloned into pCri-1a, 4a, 6a and 8a, and expressed in *E. coli* Origami 2 cells. Total (T) and soluble (S) fractions of crude protein extracts were further analysed by SDS-PAGE. All expression trials were performed at 20°C except for pCri-1a, which was also performed at 37°C. (**C**) Partially purified MBP-fragilysin before (−) and after (+) TEV proteinase cleavage. Arrows indicate the soluble fraction of fragilysin (white) and the MBP (black) after TEV proteinase cleavage. (**D**) Expression of CPA2 intracellularly (lanes 1 and 2) or periplasmatically (lanes 3 and 4) in *E. coli* cells, and extracellularly (lanes 5 and 6) in *P. pastoris* cells. Lanes indicate samples before (1, 3 and 5) and after (2, 4 and 6) tryptic digestion. Arrows indicate the pro-CPA2 (black), the mature form (grey) and the pro-peptide (white) after tryptic cleavage. (**E**) The PNGase F gene was cloned into pCri-4a and 8a and expressed overnight at 20°C in *E. coli* BL21 and Origami 2 cells. Total (T) and soluble (S) fractions of crude protein extracts were further analysed by SDS-PAGE. (**F**) Activity of affinity-purified TRX-PNGase F against glycosylated RNase B. (+) and (−) indicate presence and absence of PNGase F. Arrows indicate the PNGase F (black), native RNase B (grey) and deglycosylated RNase B (white). (**G**) MecR1 was expressed in *E. coli* BL21 using pCri-8a or 13a, and soluble fractions were analysed by Western blotting with specific antibodies as detailed in “Materials and Methods”. A black arrow indicates the detected MecR1. (**H**) Partially purified MISTIC-MecR1 after Ni-NTA-affinity purification. (**I**) Partially purified MBP-GFP, SUMO-GFP and MISTIC-MecR1 were digested with TEV proteinase, SENP1 or thrombin, respectively. For TEV proteinase and SENP1 digestions various ratios of proteinase∶tagged-protein were tested in overnight incubations at 4°C, whereas for thrombin digestions 2 units of proteinase were used to digest 25 µg of protein for various times at room temperature. Arrows indicate tagged-protein (black), target protein (grey) and fused-tag (white) after proteinase cleavage.

The pCri System allows the fusion of a His_6–8_-tag at the N-terminus of the target protein, which can be in tandem with larger tags such MBP [43], GST [65], small ubiquitin-like modifier (SUMO) [66], and the β-trefoil lectin module of protein LSL_150_ from the mushroom *Laetiporus sulphureus* (LSL) [53] (Tables 1–3). The C-terminus of the target protein can likewise be furnished with a His_6_-tag or a Strep-tag if the stop codon of the amplified gene is omitted. These tags add a functionality to the target protein, which is commonly used as a first purification step through affinity chromatography [43], [53], [65]. On this basis, we cloned and expressed GFP in pCri-1a, 4a, 6a, 8a, 11a, and 14a. The proteins were purified by Ni-NTA affinity chromatography except for MBP, GST, and LSL fusion products, which were purified by their specific affinity resins (Fig. 2A). Nickel or cobalt affinity chromatography of His_6_-tagged proteins are among the most commonly used methods for purification, but others using the affinity properties of MBP or GST, and the recently reported LSL_150_, can provide better purification results under mild elution conditions. This choice among alternative affinity purification systems allows the best purification method to be used for each target protein. Moreover, many of those tags can be used to track poorly expressed proteins by Western-blot analysis, as they are otherwise undetectable by Coomassie-stained SDS-PAGE.

#### Fusion tags assisting in protein solubility

In addition, several studies showed that tags such as N-utilisation substrate A (NUSA), MBP, or the smaller GST and SUMO have positive effects on the cargo protein due to their solubility-enhancing or chaperoning properties [2], [66], [67]. Nevertheless, their working mechanism is still controversial, with several studies suggesting a more passive role due to their excellent solubility properties rather than a direct influence on the folding of their partner [47]. For example, fragilysin (Ala212-Asp397) [59], a bacterial enterotoxin metallopeptidase, was expressed in high amounts in fusion with MBP, TRX, GST, and His_6_-tag, both at 37°C or 20°C (Fig. 2B). However, only MBP rendered the protein soluble during low temperature expression trials, whereas other fusions or expression at higher temperatures produced protein prone to aggregation. The protein remained in solution even after MBP removal (Fig. 2C) but catalytically inactive against fluorescent-labelled casein, indicating at least partial misfolding. Similar results were obtained when fragilysin was expressed with the smaller Z-tag (≈10 kDa) [59], indicating that fusion proteins may have a positive effect on target solubility without necessarily implying that it will be well folded and active. Nevertheless, these fusion tags can have an application in the expression of proteins with known solubility problems that need to be temporally stabilised until an adequate condition/solution is found [67].

#### Expression of proteins requiring disulfide bonds and other posttranslational modifications

Correct folding and stabilization requires the formation of disulfide bonds in many proteins. These can be formed in oxidising environments as found in the periplasmic and extracellular environment of bacteria, or in specialised organelles of eukaryotes. *B. subtilis* has a large secretory capacity, whereas in *E. coli* secretion is mainly limited to the periplasm [68], [69]. In *P. pastoris*, proteins are first driven to the endoplasmic reticulum and, after folding, they are secreted to the extracellular medium [70]. The pCri System includes vectors that fuse SP specialised for protein translocation to these cellular compartments. pCri-9 and 12 can be used with *E. coli* cells, whereas pCri-16 and 18 are suitable for expression in *P. pastoris* and *B. subtilis*, respectively. In the case of pCri-12, a disulfide-bond isomerase C (DsbC) is coexpressed with the target protein and provides additional support in the correct pairing of disulfide bonds in the periplasm [50].

As a test protein, we used human CPA2, which is commonly expressed in *P. pastoris* cells [71]. Unexpectedly, expression trials indicated that the protein is produced not only in the extracellular environment of *P. pastoris* but also in the cytoplasm and periplasm of *E. coli* cells (Fig. 2D). In contrast, *B. subtilis* did not express the protein either extracellularly or intracellularly. In all cases, the protein was soluble and correctly processed after limited tryptic digestion, showing activity against small substrates. However, this is not always the case. Besides the oxidising conditions other proteins may often participate in correct folding, including oxidases, foldases, isomerases and specialised chaperones [69]. Moreover, disulfide bond formation is not the only factor in proper protein folding and stability, and further posttranslational modifications (e.g. glycosylation) may be required, which can be provided by *P. pastoris* [70].

Another approach for disulfide bond formation exploits the oxidising cytoplasm of thioredoxin reductase B (*trx*B^−^) and glutathione reductase (*gor* ^−^) mutant *E. coli* cells (Origami 2) [34]. In contrast to the commonly used BL21 cells, Origami 2 efficiently expressed PNGase F, either with pCri-4a or 8a, soluble and catalytically active against RNase B (Fig. 2E and 2F). The protein contains disulfide bonds that require an oxidising environment, which is adequately formed in the cytoplasm of mutant cells. In addition, the combined use of thioredoxin A (TRX) as fusion protein in pCri-4a and expression in Origami 2 can lead to the overexpression of small multi-disulfide proteins, among others [69], [72]. This system takes advantage of TRX, which acts as an oxidant when it operates in an oxidized milieu found in mutant cells [34], thus providing an additional mechanism for disulfide bond formation within the cytoplasm. TRX is subsequently removed by TEV proteinase cleavage in the presence of selected amounts of redox agents to assist in correct disulfide bond pairing [72], [73].

#### Expression of membrane proteins

Membrane proteins are among the targets most requested and at the same time difficult to express and purify. To address this issue, a vector was prepared, which fuses a small protein from *B. subtilis* with target proteins (pCri-13a). This protein, known as the membrane-integrating sequence for translation of integral-membrane protein constructs (MISTIC), folds autonomously into membranes, simultaneously dragging the tagged-protein to the cell membrane [74]. Moreover, this vector contains a longer His_8_-tag in tandem with MISTIC in order to provide higher affinity for Ni-NTA affinity purification.

For evaluation purposes, we cloned and expressed MecR1, a membrane metallopeptidase from *Staphylococcus aureus* implicated in methicillin resistance [75]. Detectable levels of expression were only achieved when the protein was fused with MISTIC, whereas mere fusion with an N-terminal His_6_-tag was unsuccessful (Fig. 2G). Moreover, expression yields of the protein were sufficient (0.4 mg of affinity purified protein per litre of culture) to enable partial purification by Ni-NTA affinity chromatography after solubilisation of the membranes with the zwitterionic detergent LDAO (Fig. 2H). Although further studies are required to assess the folding state of this protein, fusion with MISTIC allowed us to express it in milligram amounts. Many other membrane proteins were also expressed in fusion with various MISTIC constructs, indicating that this system could be an alternative approach for membrane proteins that are difficult to express [52].

#### Removal of fusion tags

In most cases, release of the target protein from any fused tag is desirable. In the pCri System, a TEV proteinase cleavage site is introduced immediately after the tag in all vectors except for pCri-11 and pCri-13, in which a SENP1 or thrombin site is found, respectively (Fig. S1). TEV proteinase is a highly specific enzyme that recognises an hexapeptide sequence [76], whereas SENP1 further offers robustness and high proteolytic activity in addition to high specificity, usually requiring only minute amounts for tag removal [77]. Moreover, use of a thrombin cleavage site in pCri-13a was necessary due to the low efficiency of TEV proteinase in the presence of detergents which are required during membrane-protein solubilisation [78]. In addition, linker sequences (Gly-Ser)_5_ and Gly_3_-Ala were introduced before and after the thrombin recognition site, respectively, to improve access for proteinase cleavage (Fig. S1) [79].

Tag removal was achieved with variable amounts of endopeptidases, different incubation times and temperatures (Fig. 2I). These studies indicated that optimisation trials are needed in each case to identify the best conditions for complete digestion (e.g. buffer, temperature, proteinase∶substrate ratio). Proteinase cleavage and tag removal result in the incorporation of one or two extra residues at the N-terminus of the expressed protein except for pCri-4b and pCri-13a, which attach three and six residues, respectively (Tables 1–3).

## Conclusions

Here we introduce a vector collection designed for large-scale recombinant protein overexpression, and demonstrate its suitability in a series of test proteins. The choice of a suitable expression vector should be based on target and tag properties. The availability of a range of fusion tags allows the choice between different affinity purification methods. Moreover, some tags were included for specific use, such as MISTIC and TRX, which are intended for expression of membrane and disulfide rich proteins, respectively. In general, our common strategy first explores the effects of the presence or absence of N- or C- terminal tags (e.g. His_6_-tag or Strep-tag) on each construct under different host cell growth conditions. Omission of the tag or alternation of the position can drastically influence the expression and solubility of the protein. If this approach is ineffective the chances of optimising the expression by testing other fusion combinations are reduced. Several reports showed the beneficial effects of the fusions on target solubility [2], [67]. However, this is not always the case: the protein is often dragged into solution, rather than acting as a chaperone for the proper folding of its fusion partner. Removal of the fusion tag can revert the positive effect and cause precipitation [47], [48]. If this occurs, then modified constructs, other homologous targets or even other expression systems need to be explored, including bacterial and eukaryotic cells that can be easily tested using the vector collection of the pCri System.

## Supporting Information

Figure S1 **Partial nucleotide sequence and translation of the pCri System vectors.**
(DOC)
