# Supplementary material for: The pCri System: A Vector Collection for Recombinant Protein Expression and Purification
Source: PLoS One. 2014 Nov 11;9(11):e112643. doi: 10.1371/journal.pone.0112643 (PMC4227841; doi:10.1371/journal.pone.0112643)
Supplement: Figure S1 — Partial nucleotide sequence and translation of the pCri System vectors. (DOC) [file pone.0112643.s001.doc]

**Supporting Information.**

**Figure S1.** Partial nucleotide sequence and translation of the pCri System vectors.
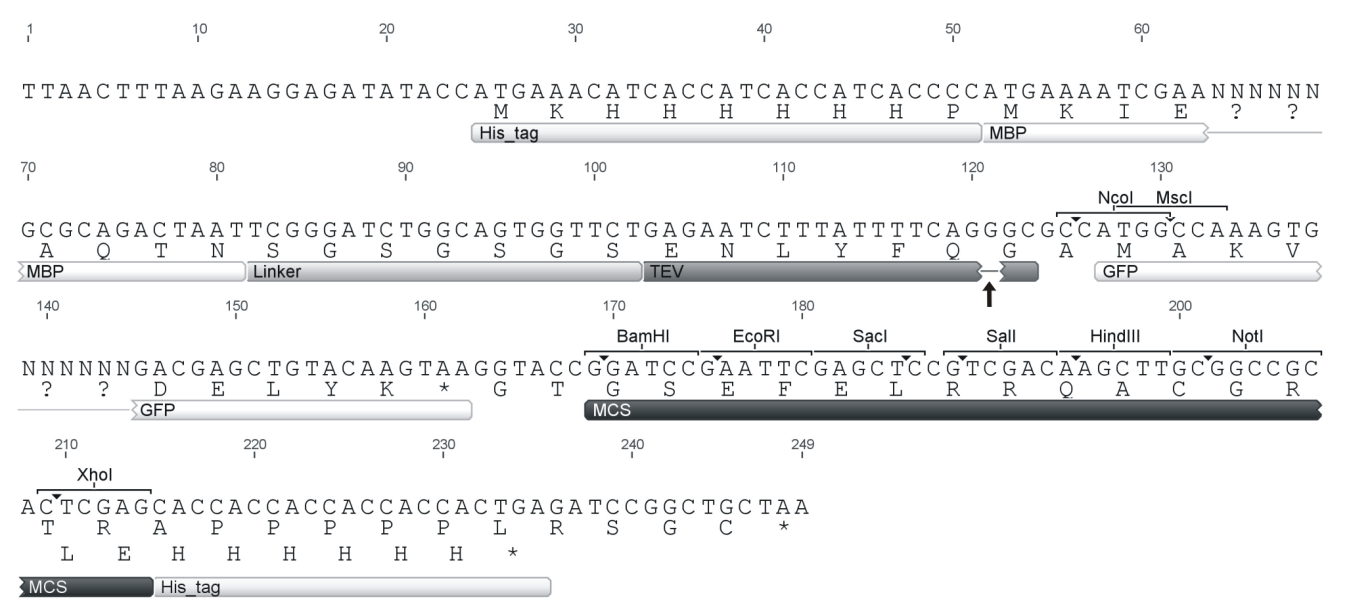


**pCri-1a map.** Nucleotide sequence and translation of the vector cloning site. Parts of the MBP and GFP sequences that are omitted from the vector map are marked by (?) and (N) symbols. A black arrow indicates the TEV proteinase cleavage site. The restriction sites shown are found once.


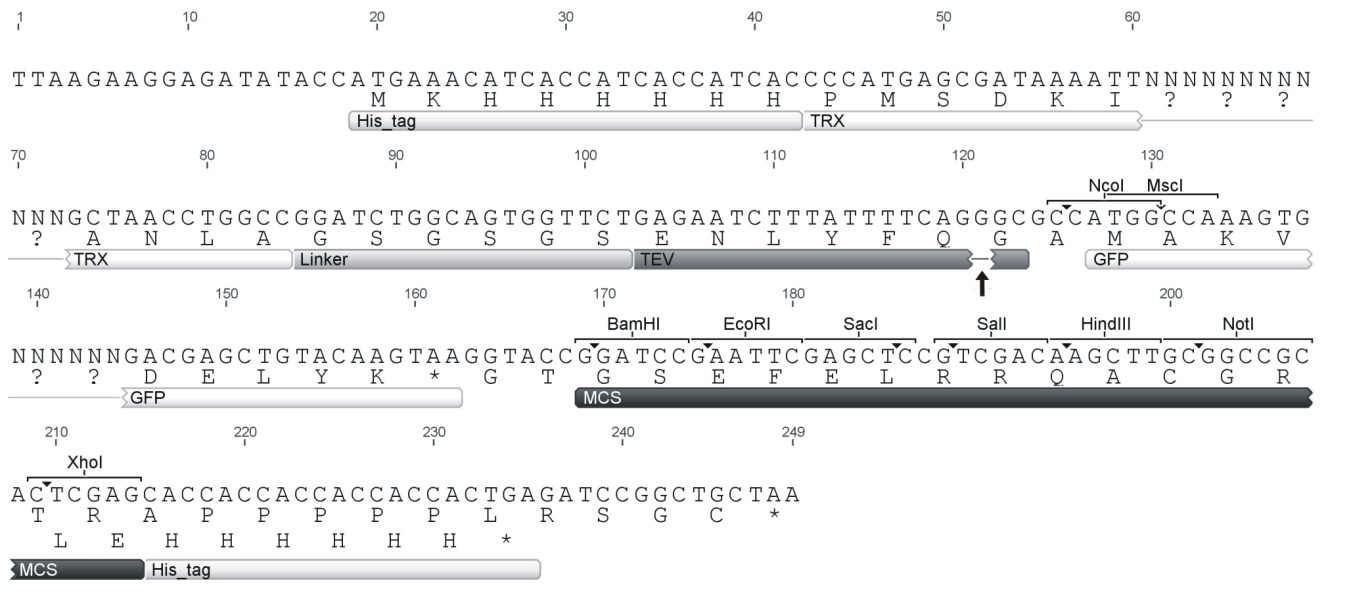


**pCri-4a map.** Nucleotide sequence and translation of the vector cloning site. Parts of the TRX and GFP sequences that are omitted from the vector map are marked by (?) and (N) symbols. A black arrow indicates the TEV proteinase cleavage site. The restriction sites shown are found once.


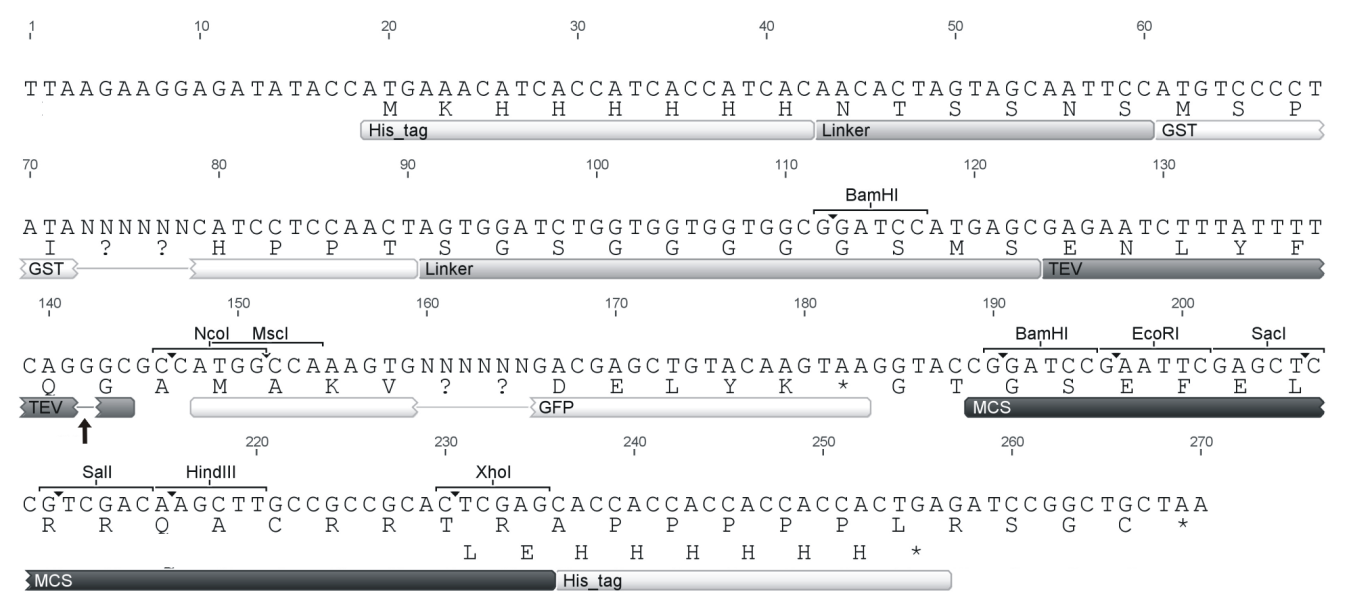


**pCri-6a map.** Nucleotide sequence and translation of the vector cloning site. Parts of the GST and GFP sequences that are omitted from the vector map are marked by (?) and (N) symbols. A black arrow indicates the TEV cleavage site. The restriction sites shown are found once except for *Msc*I and *Bam*HI, which are alsowithin the GST nucleotide sequence.


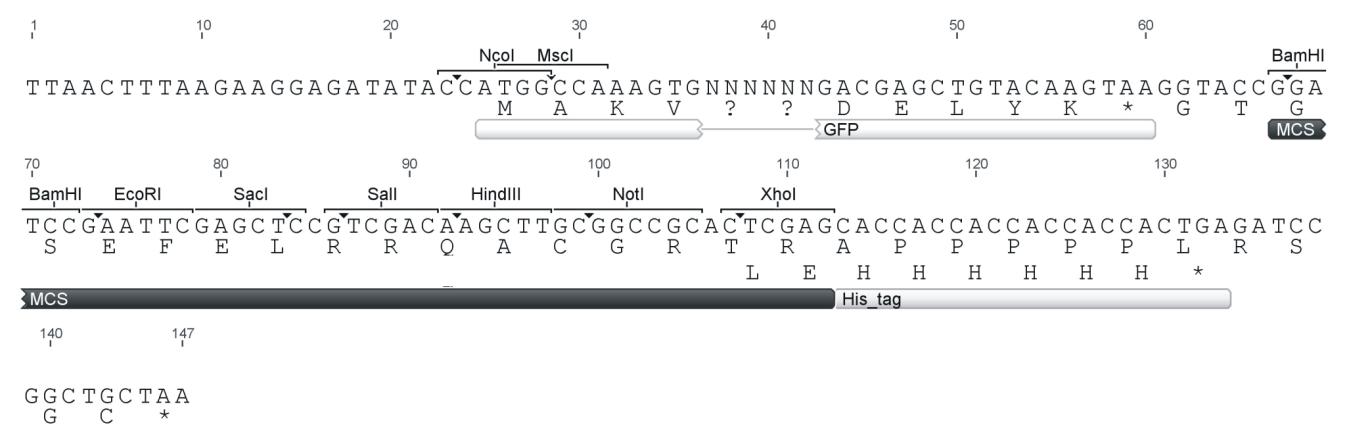


**pCri-7a map.** Nucleotide sequence and translation of the vector cloning site. Part of the GFP sequence that is omitted from the vector map are marked by (?) and (N) symbols. The restriction sites shown are found once.


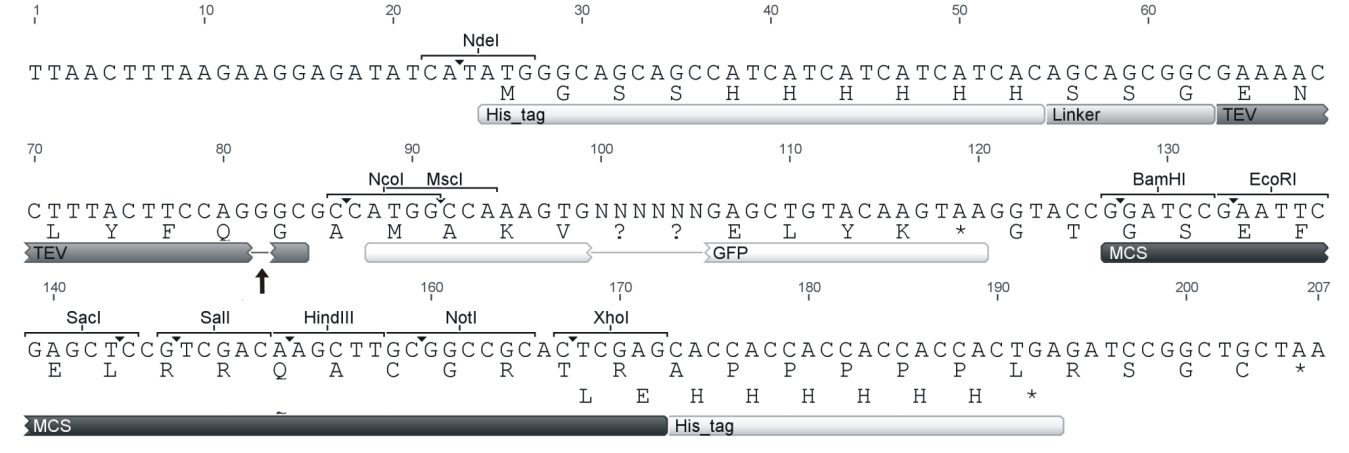


**pCri-8a map.** Nucleotide sequence and translation of the vector cloning site. Part of the GFP sequence that is omitted from the vector map are marked by (?) and (N) symbols. A black arrow indicates the TEV proteinase cleavage site. The restriction sites shown are found once.


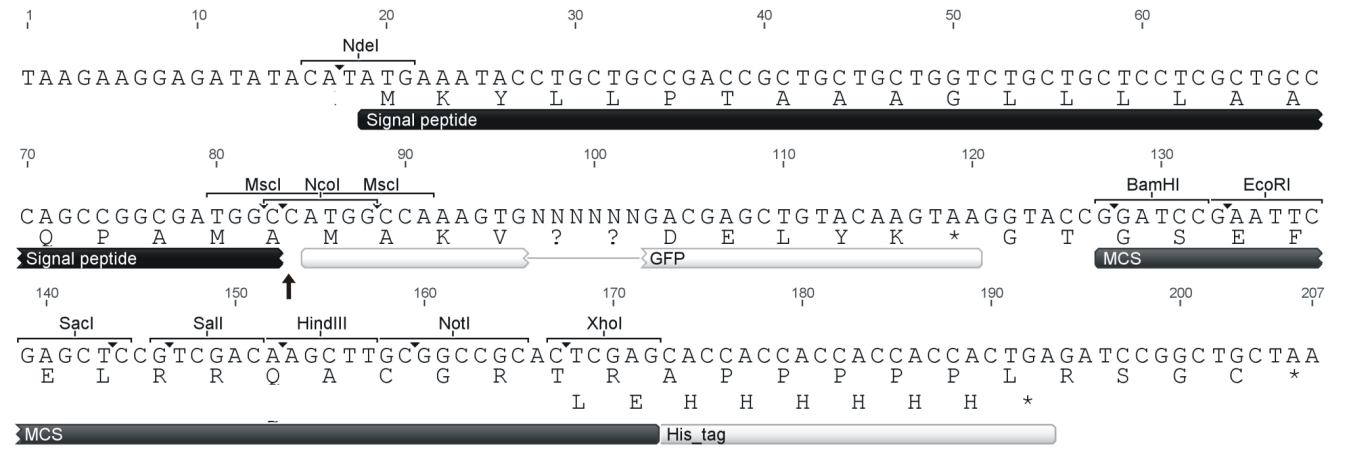


**pCri-9a map.** Nucleotide sequence and translation of the vector cloning site. Part of the GFP sequence that is omitted from the vector map are marked by (?) and (N) symbols. A black arrow indicates the SP cleavage site. The restriction sites shown are found once.


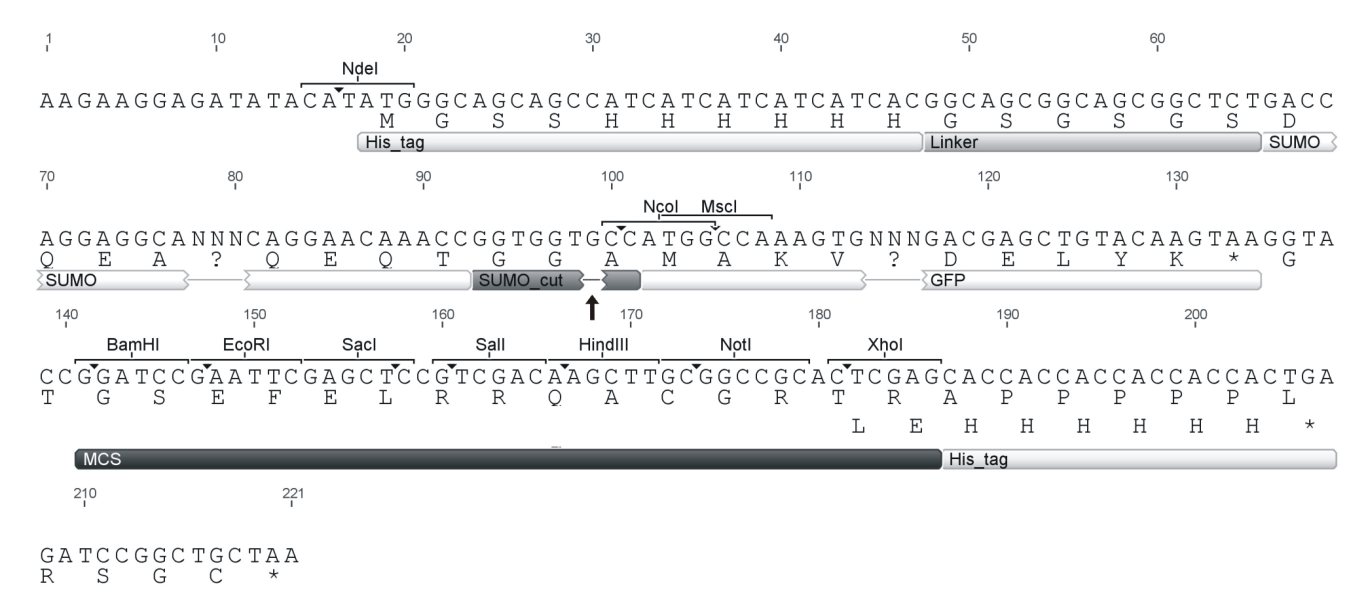


**pCri-11a map.** Nucleotide sequence and translation of the vector cloning site. Parts of the SUMO and GFP sequences that are omitted from the vector map are marked by (?) and (N) symbols. A black arrow indicates the SENP1 cleavage site. The restriction sites shown are found once except for *Eco*RI, which is also within the SUMO nucleotide sequence.


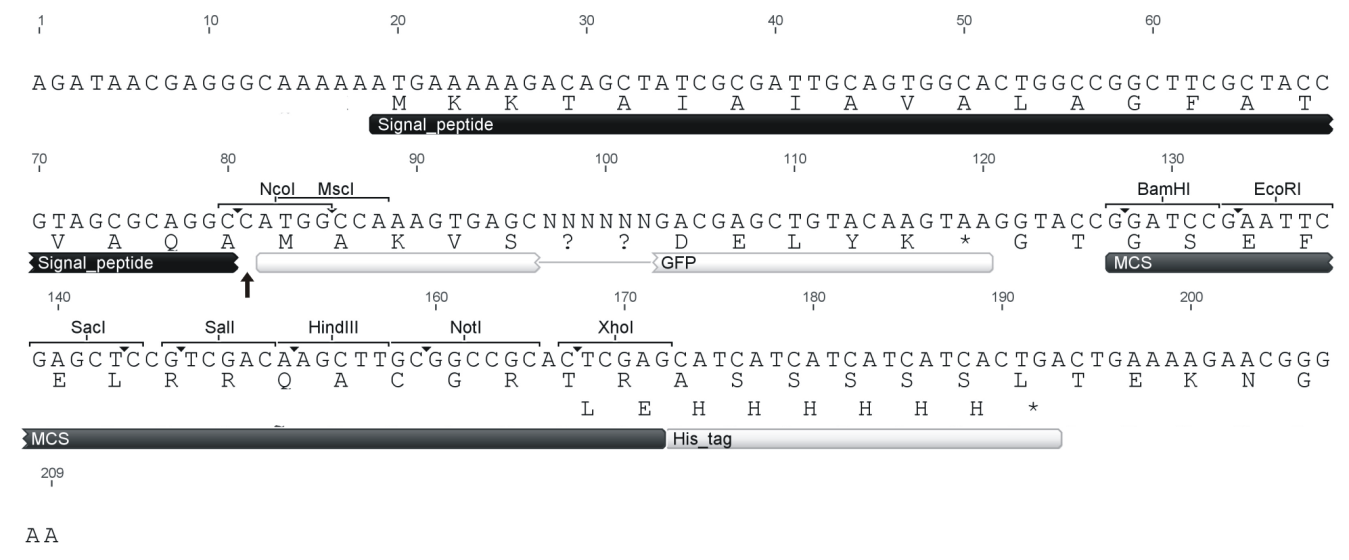


**pCri-12a map.** Nucleotide sequence and translation of the vector cloning site. Part of the GFP sequence that is omitted from the vector map are marked by (?) and (N) symbols. A black arrow indicates the SP cleavage site. The restriction sites shown are found once except for *Bam*HI, *Eco*RI and *Hind*III, which are also elsewhere within the nucleotide sequence.

**
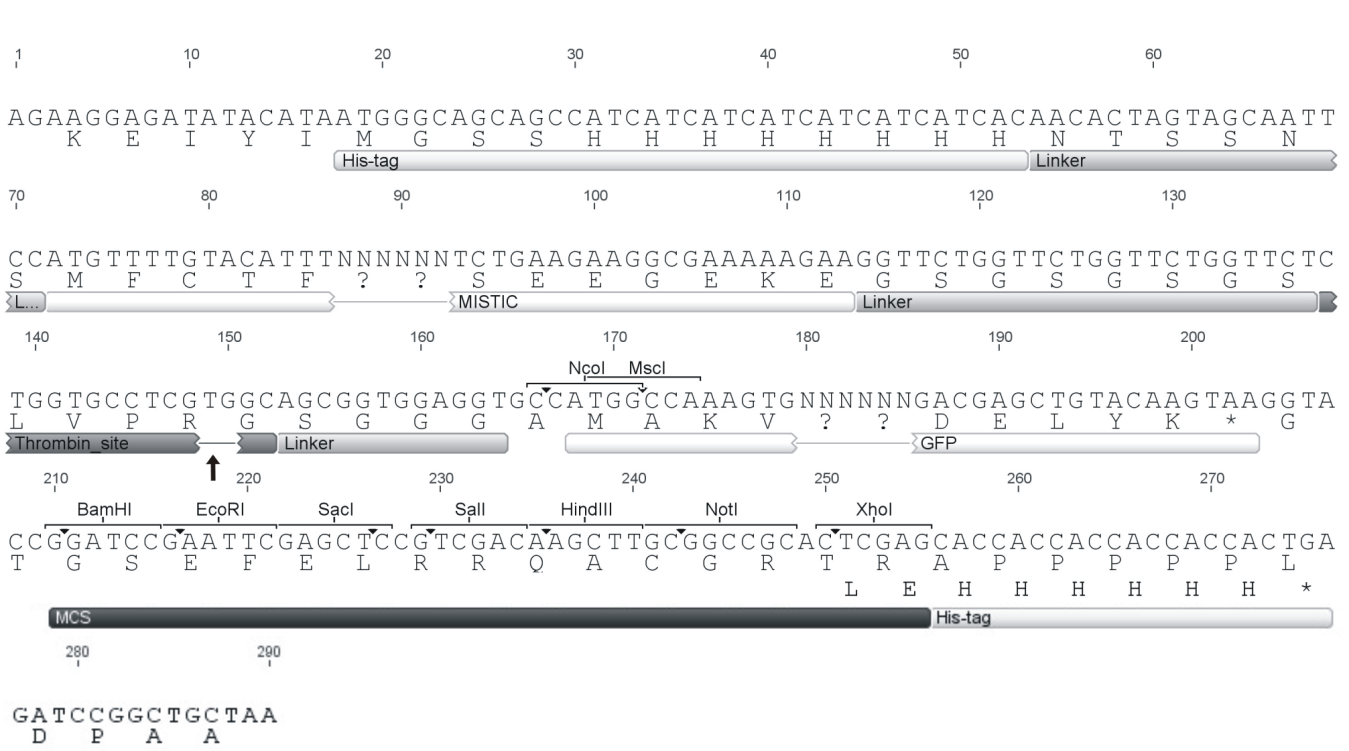
**

**pCri-13a map.** Nucleotide sequence and translation of the vector cloning site. Part of the MISTIC and GFP sequence that are omitted from the vector map are marked by (?) and (N) symbols. A black arrow indicates the thrombin cleavage site. The restriction sites shown are found once.


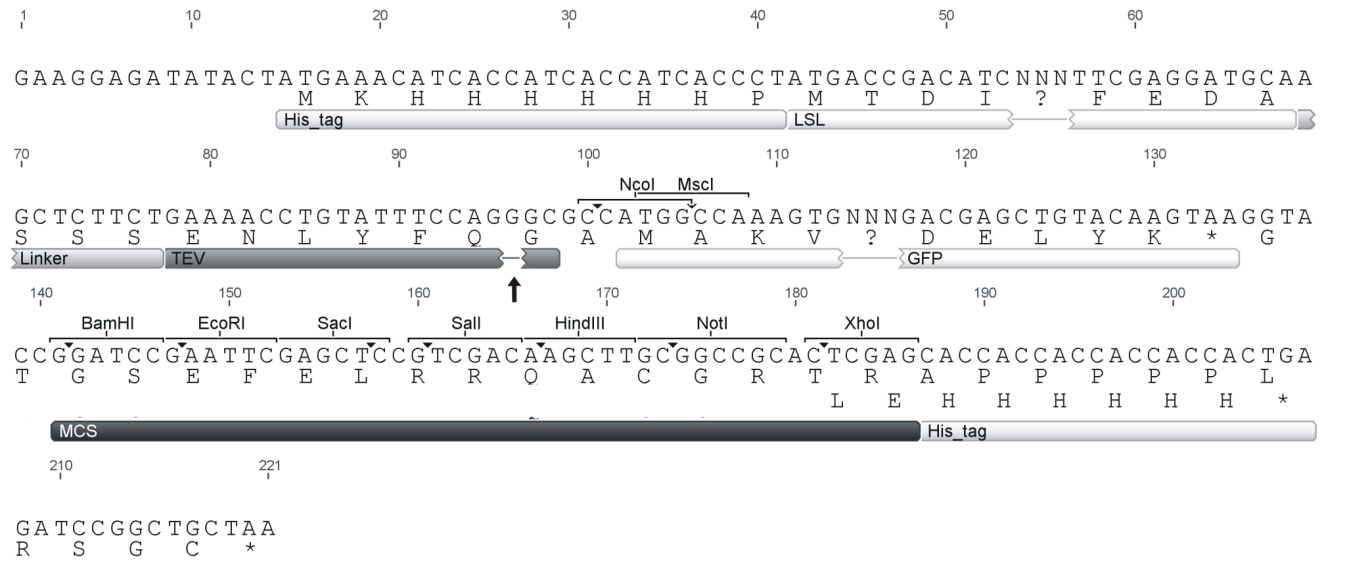


**pCri-14a map.** Nucleotide sequence and translation of the vector cloning site. Parts of the LSL and GFP sequences that are omitted from the vector map are marked by (?) and (N) symbols. A black arrow indicates the TEV proteinase cleavage site. The restriction sites shown are found once except for *Sal*I, which is also within the LSL nucleotide sequence.

**
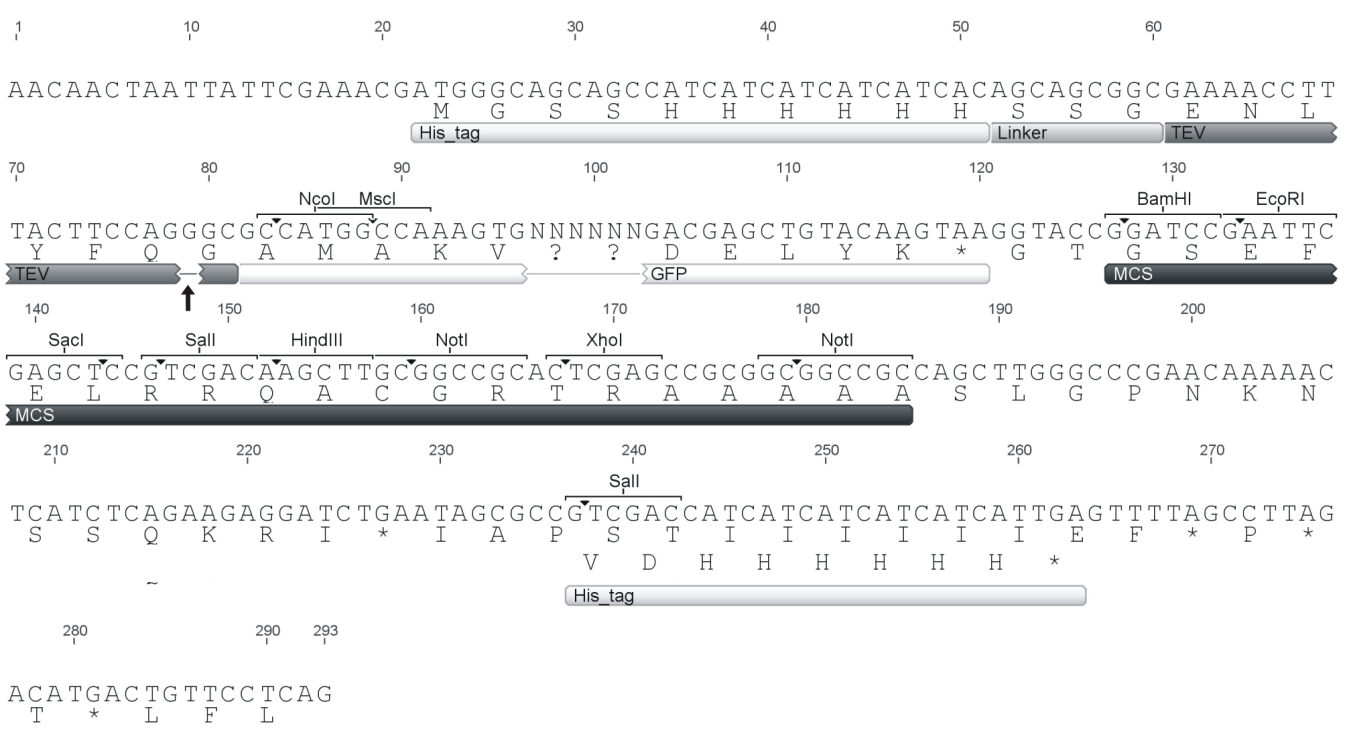
**

**pCri-15a map.** Nucleotide sequence and translation of the vector cloning site. Part of the GFP sequence that is omitted from the vector map are marked by (?) and (N) symbols. A black arrow indicates the TEV proteinase cleavage site. The restriction sites shown are found once except of *Bam*HI, *Hind*III, *Sac*I and *Msc*I which are also elsewhere within the nucleotide sequence.


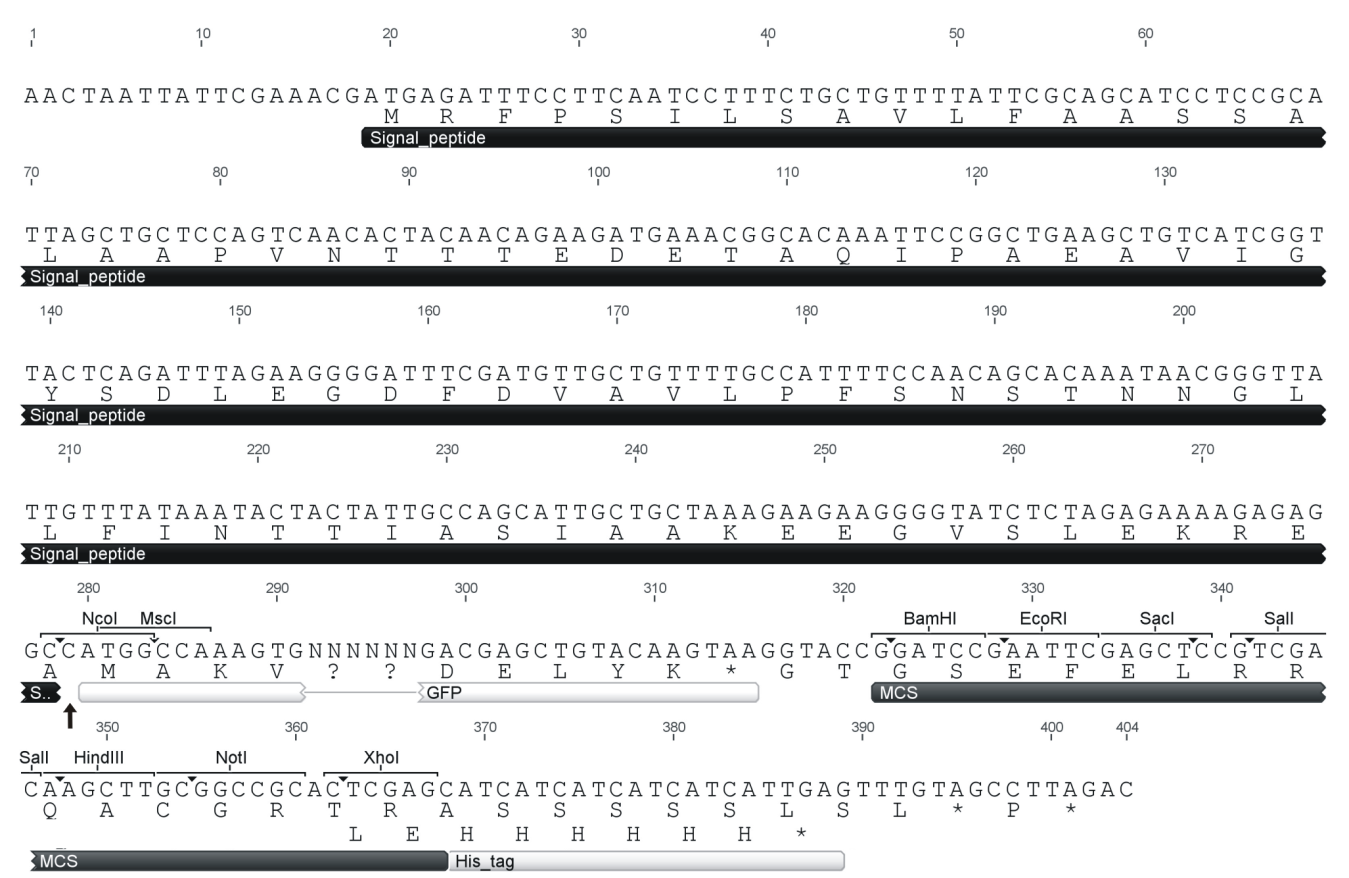


**pCri-16a map.** Nucleotide sequence and translation of the vector cloning site. Part of the GFP sequence that is omitted from the vector map are marked by (?) and (N) symbols. A black arrow indicates the SP cleavage site. The restriction sites shown are found once except for *Bam*HI, *Hind*III, *Sac*I and *Msc*I, which are also elsewhere within the nucleotide sequence.


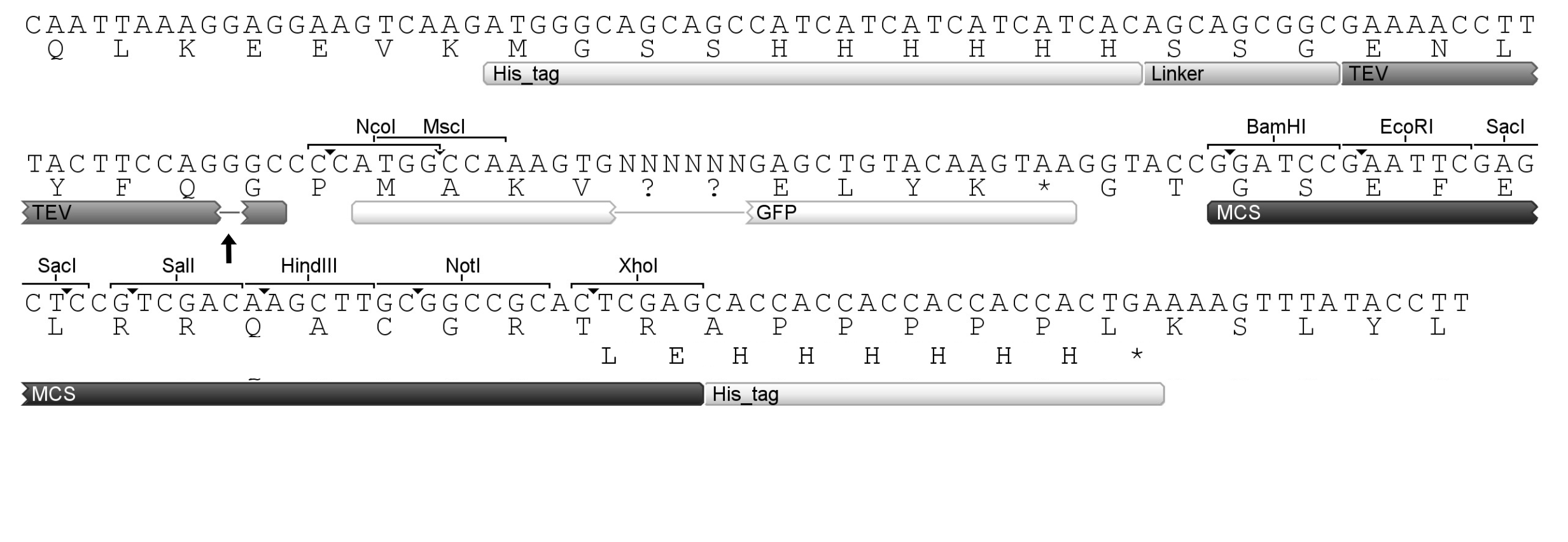
**pCri-17a map.** Nucleotide sequence and translation of the vector cloning site. Part of the GFP sequence that is omitted from the vector map are marked by (?) and (N) symbols. A black arrow indicates the TEV proteinase cleavage site. The restriction sites shown are found once except for *Eco*RI, *Sac*I, *Bam*HI, *Sal*I, and *Hind*III, which are also elsewhere within the nucleotide sequence.


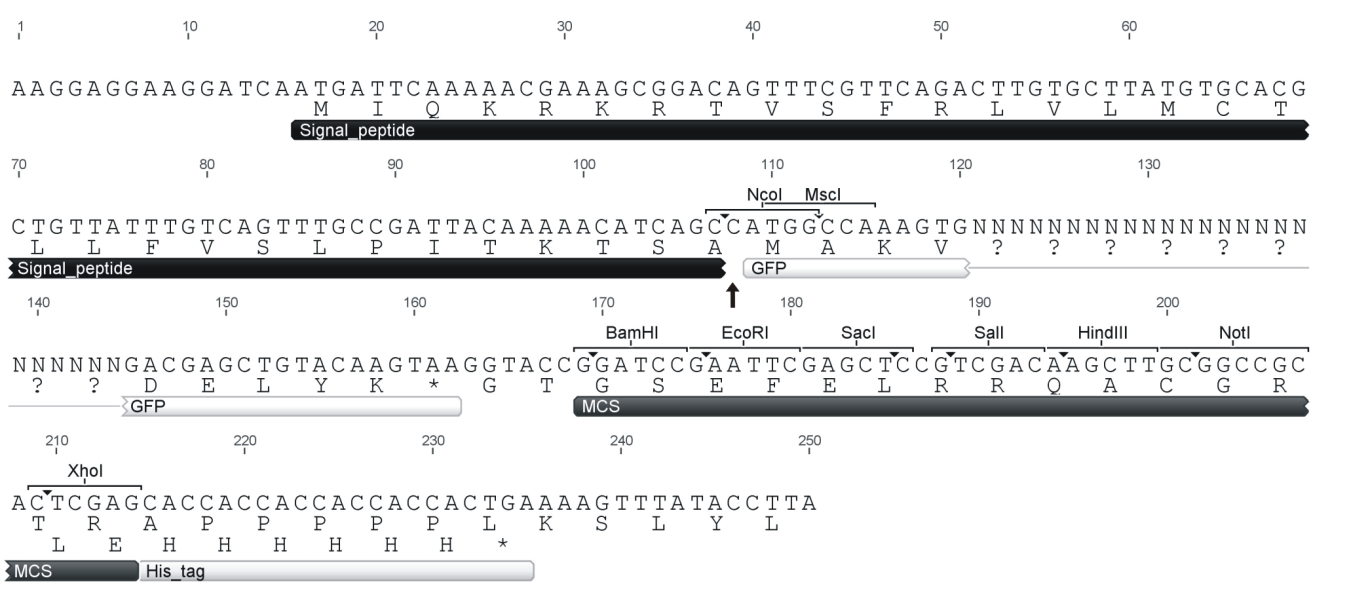


**pCri-18a map.** Nucleotide sequence and translation of the vector cloning site. Part of the GFP sequence that is omitted from the vector map are marked by (?) and (N) symbols. A black arrow indicates the SP cleavage site. The restriction sites shown are found once except for *Eco*RI, *Sac*I, *Bam*HI, *Sal*I, and *Hind*III, which are also elsewhere within the nucleotide sequence.


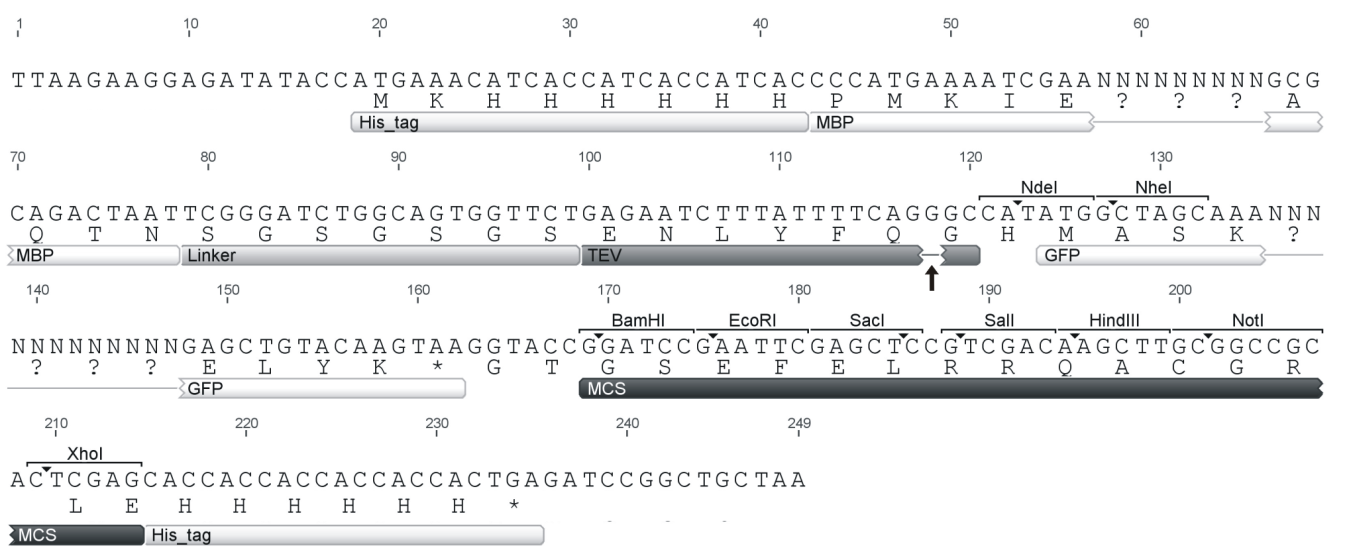


**pCri-1b map.** Nucleotide sequence and translation of the vector cloning site. Parts of the MBP and GFP sites that are omitted from the vector map are marked by (?) and (N) symbols. A black arrow indicates the TEV proteinase cleavage site. The restriction sites shown are found once.


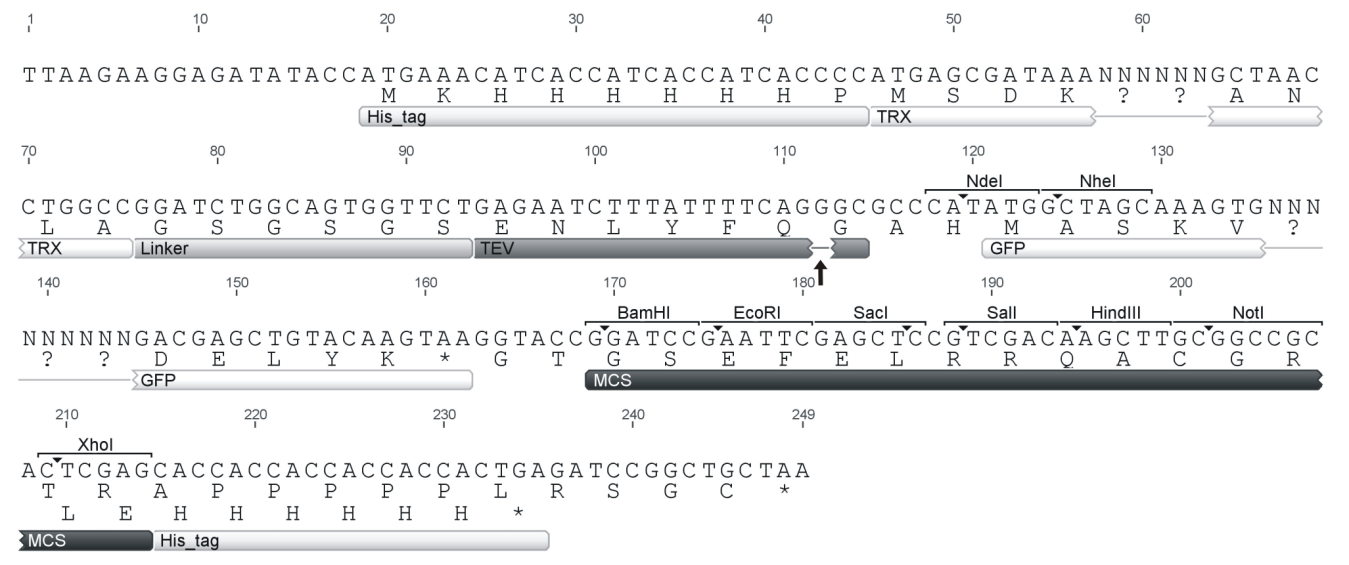


**pCri-4b map.** Nucleotide sequence and translation of the vector cloning site. Parts of the TRX and GFP sequences that are omitted from the vector map are marked by (?) and (N) symbols. A black arrow indicates the TEV proteinase cleavage site. The restriction sites shown are found once.


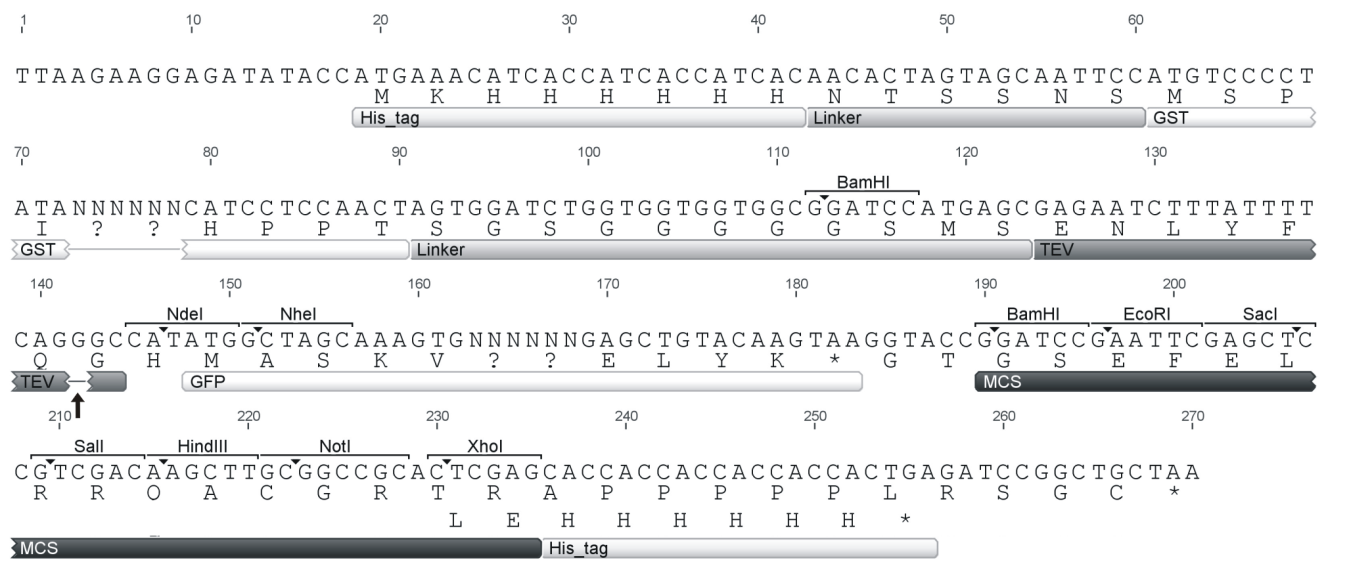


**pCri-6b map.** Nucleotide sequence and translation of the vector cloning site. Part of the GST and GFP sequences that are omitted from the vector map are marked by (?) and (N) symbols. A black arrow indicates the TEV proteinase cleavage site. The restriction sites shown are found once except for *Msc*I and *Bam*HI, which are also within the GST nucleotide sequence.


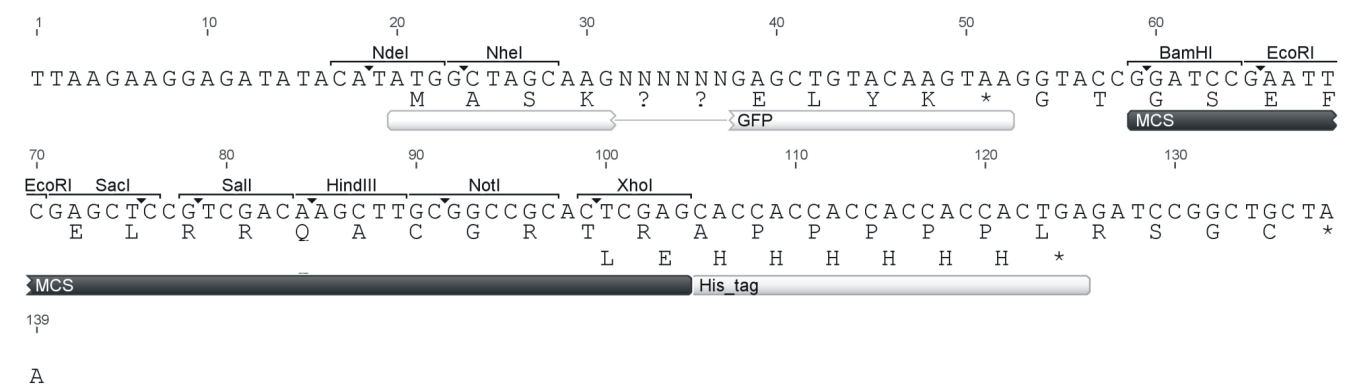


**pCri-7b map.** Nucleotide sequence and translation of the vector cloning site. Part of the GFP sequence that is omitted from the vector map are marked by (?) and (N) symbols. The restriction sites shown are found once.


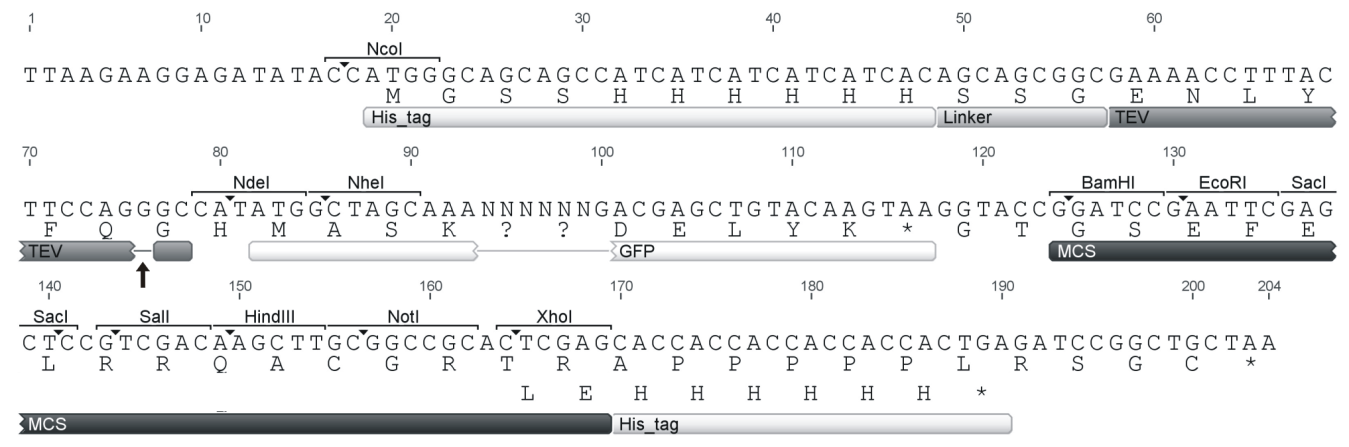


**pCri-8b map.** Nucleotide sequence and translation of the vector cloning site. Part of the GFP sequence that is omitted from the vector map are marked by (?) and (N) symbols. A black arrow indicates the TEV proteinase cleavage site. The restriction sites shown are found once.


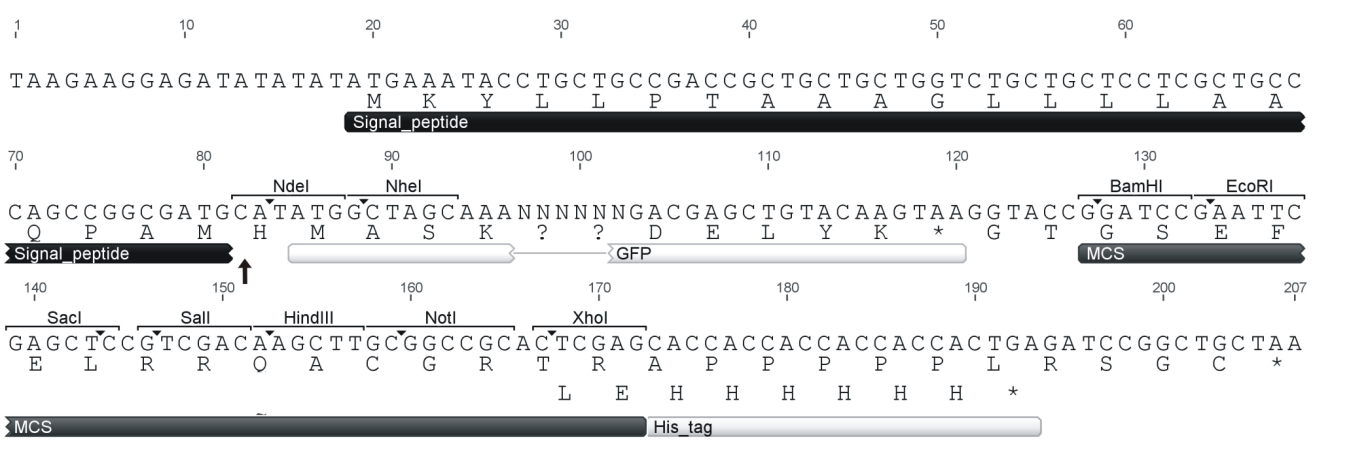


**pCri-9b map.** Nucleotide sequence and translation of the vector cloning site. Part of the GFP sequence that is omitted from the vector map are marked by (?) and (N) symbols. A black arrow indicates the SP cleavage site. The restriction sites shown are found once.


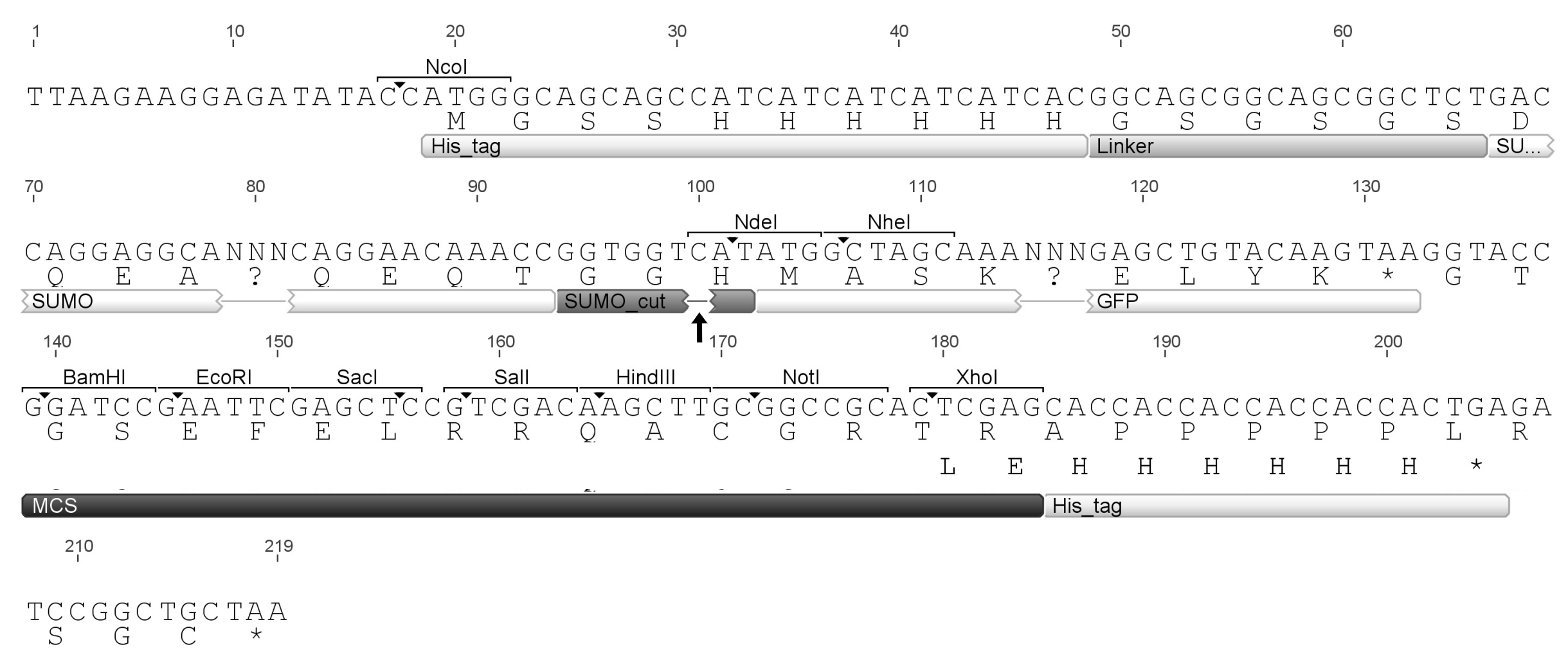


**pCri-11b map.** Nucleotide sequence and translation of the vector cloning site. Parts of the SUMO and GFP sequences that are omitted from the vector map are marked by (?) and (N) symbols. A black arrow indicates the SENP1 cleavage site. The restriction sites shown are found once except for *Eco*RI, which is also within the SUMO nucleotide sequence.


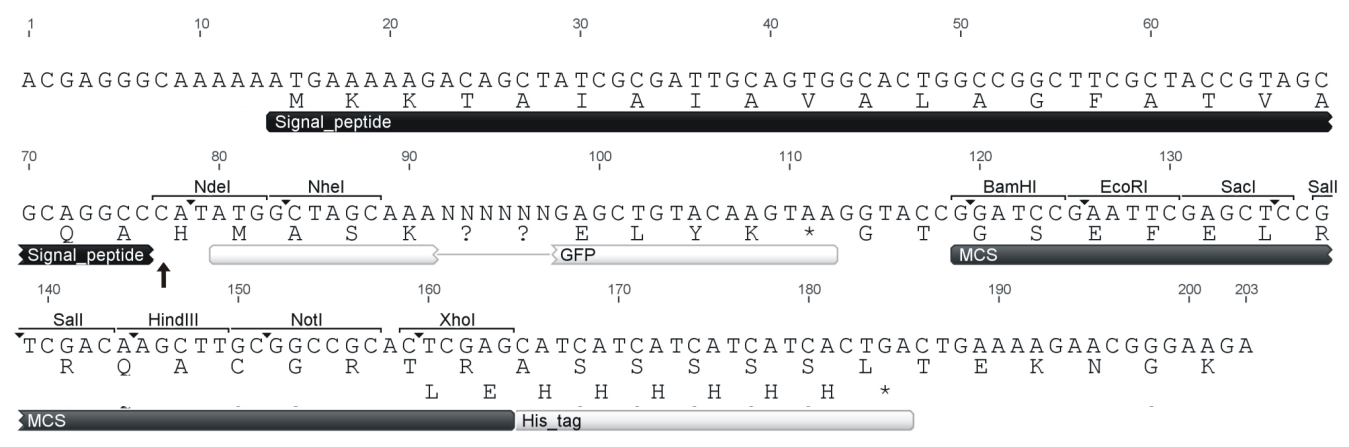


**pCri-12b map.** Nucleotide sequence and translation of the vector cloning site. Part of the GFP sequence that is omitted from the vector map are marked by (?) and (N) symbols. A black arrow indicates the SP cleavage site. The restriction sites shown are found once except for *Bam*HI, *Eco*RI and *Hind*III, which are also elsewhere within the nucleotide sequence.


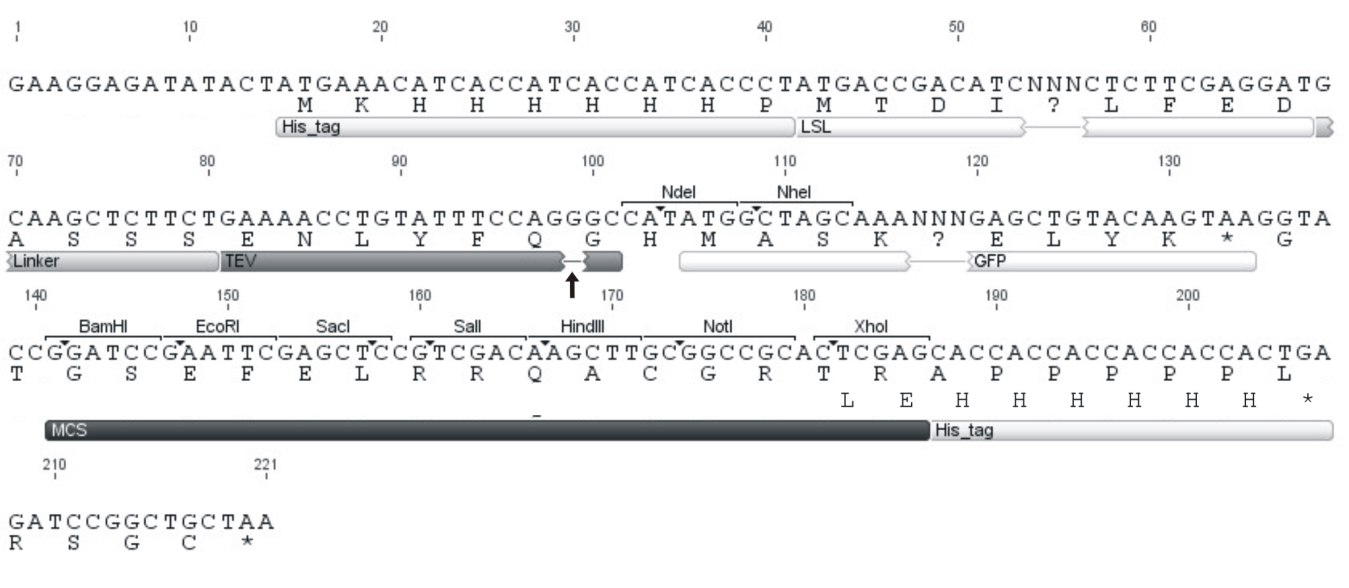
**pCri-14b map.** Nucleotide sequence and translation of the vector cloning site. Parts of the LSL and GFP sequences that are omitted from the vector map are marked by (?) and (N) symbols. A black arrow indicates the TEV proteinase cleavage site. The restriction sites shown are found once except for *Sal*I, which is also within the LSL nucleotide sequence.


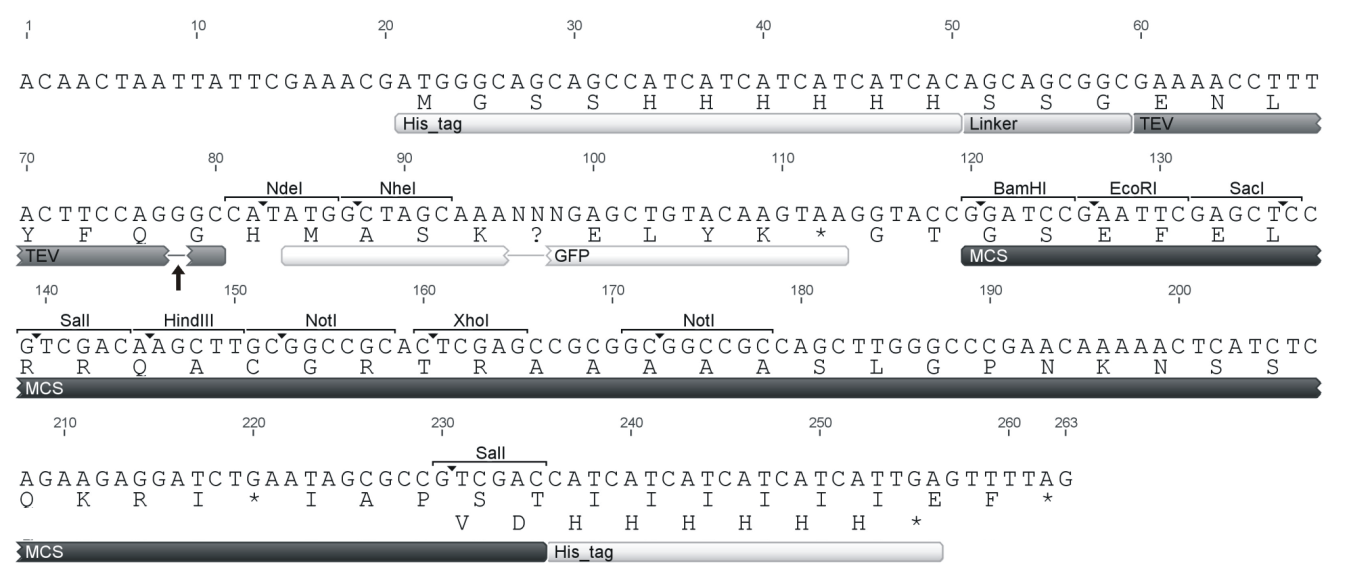


**pCri-15b map.** Nucleotide sequence and translation of the vector cloning site. Part of the GFP sequence that is omitted from the vector map are marked by (?) and (N) symbols. A black arrow indicates the TEV proteinase cleavage site. The restriction sites shown are found once except for *Bam*HI, *Hind*III, *Sac*I, and *Msc*I, which are also elsewhere within the nucleotide sequence.


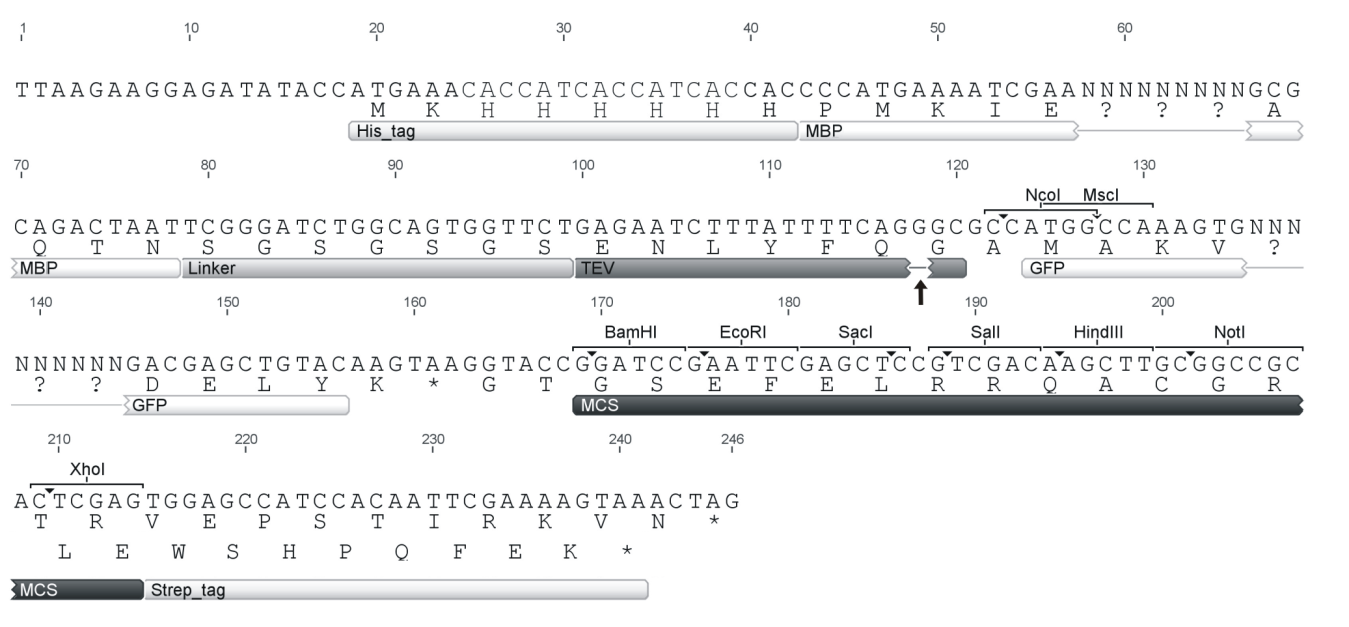


**pCri-1a-Strep map.** Nucleotide sequence and translation of the vector cloning site. Parts of the MBP and GFP sites that are omitted from the vector map are marked by (?) and (N) symbols. A black arrow indicates the TEV proteinase cleavage site. The restriction sites shown are found once.


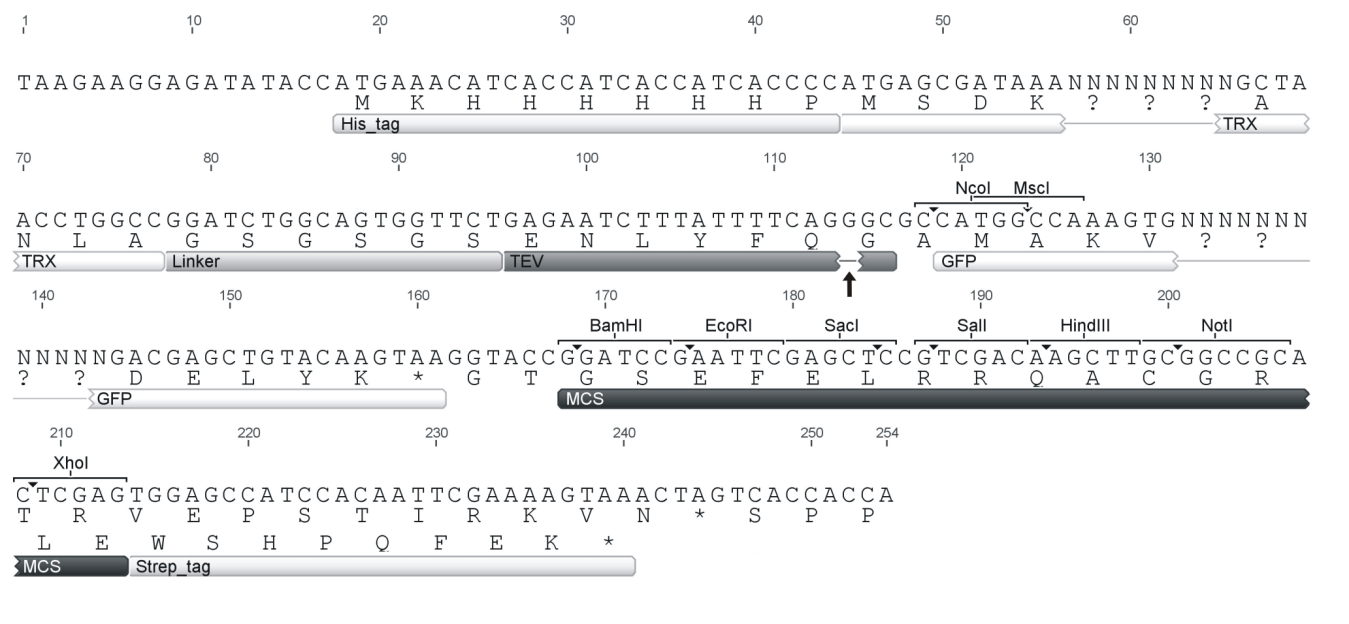


**pCri-4a-Strep map.** Nucleotide sequence and translation of the vector cloning site. Parts of the TRX and GFP sequences that are omitted from the vector map are marked by (?) and (N) symbols. A black arrow indicates the TEV proteinase cleavage site. The restriction sites shown are found once.


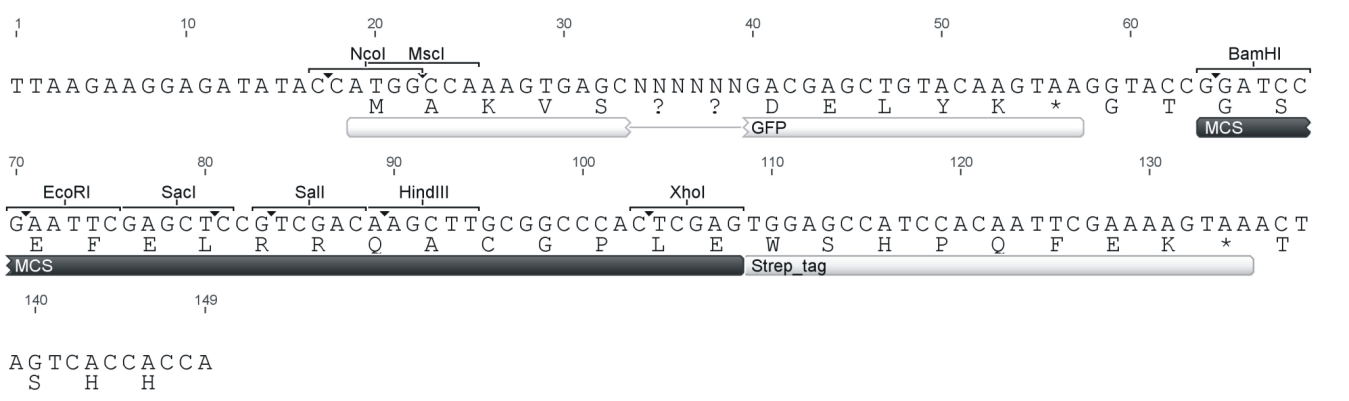


**pCri-7a-Strep map.** Nucleotide sequence and translation of the vector cloning site. Part of the GFP sequence that is omitted from the vector map is marked by (?) and (N). The restriction sites shown are found once.


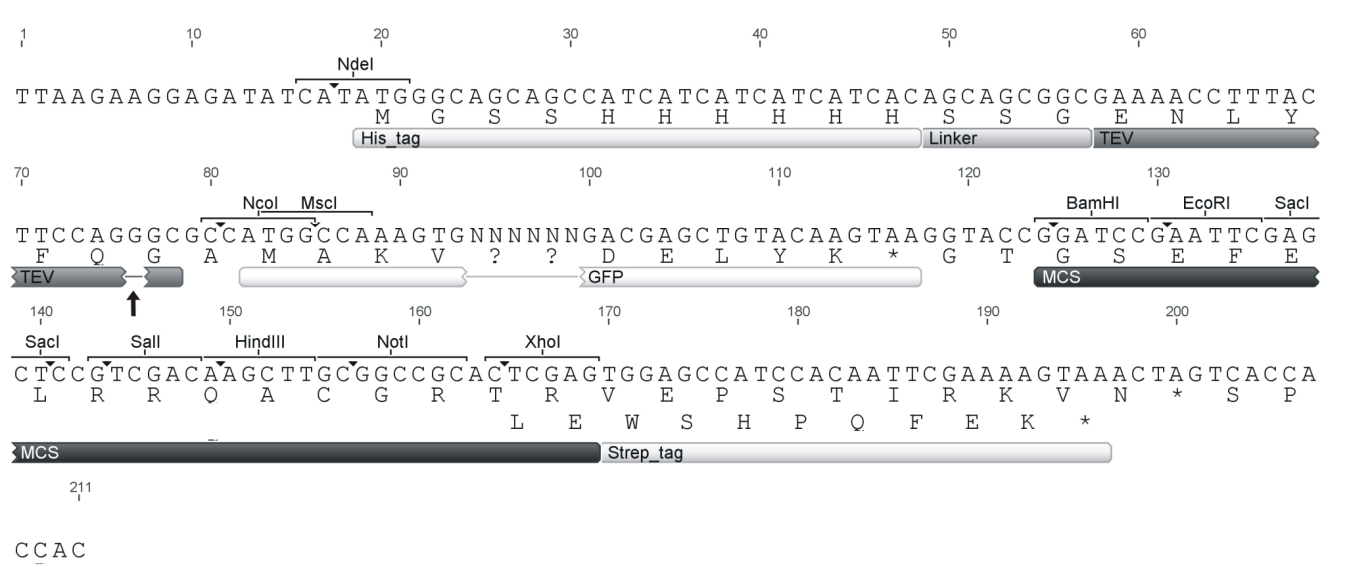


**pCri-8a-Strep map.** Nucleotide sequence and translation of the vector cloning site. Part of the GFP sequence that is omitted from the vector map are marked by (?) and (N). A black arrow indicates the TEV proteinase cleavage site. The restriction sites shown are found once.


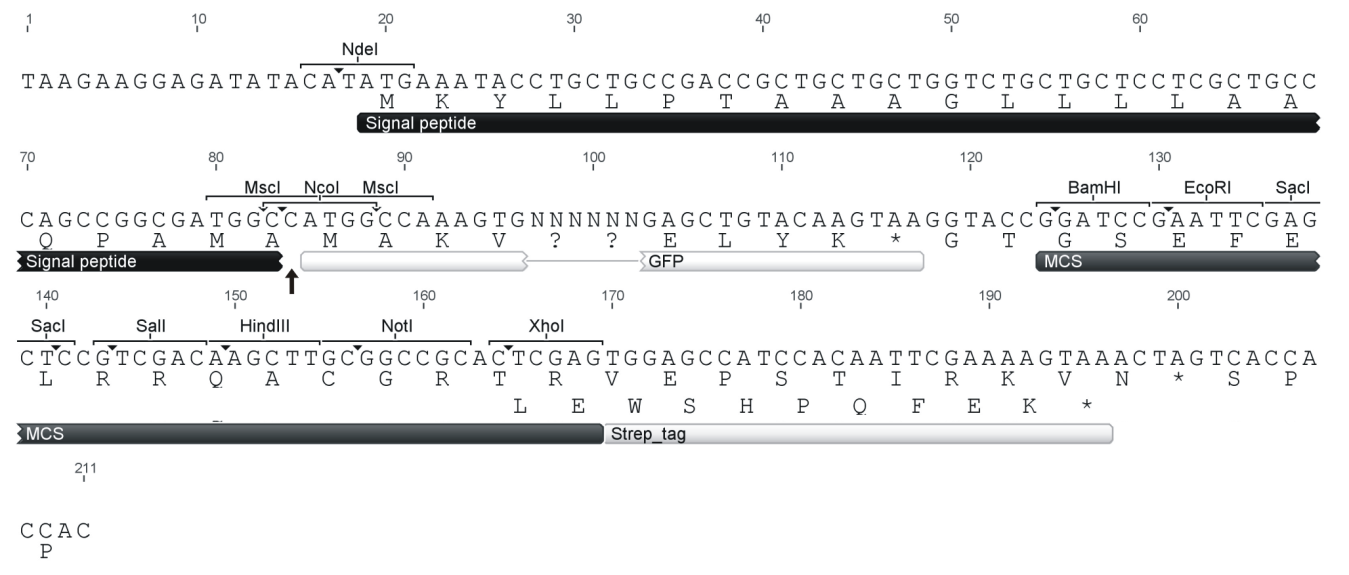


**pCri-9a-Strep map.** Nucleotide sequence and translation of the vector cloning site. Part of the GFP sequence that is omitted from the vector map are marked by (?) and (N) symbols. A black arrow indicates the SP cleavage site. The restrictions sites shown are found once.
